# miR-125a-5p is a prognostic biomarker that targets HDAC4 to suppress breast tumorigenesis

**DOI:** 10.18632/oncotarget.2674

**Published:** 2014-11-28

**Authors:** Tsung-Hua Hsieh, Chia-Yi Hsu, Cheng-Fang Tsai, Cheng-Yu Long, Chee-Yin Chai, Ming-Feng Hou, Jau-Nan Lee, Deng-Chyang Wu, Shao-Chun Wang, Eing-Mei Tsai

**Affiliations:** ^1^ Graduate Institute of Medicine, College of Medicine, Kaohsiung Medical University, Kaohsiung 80708, Taiwan; ^2^ Center for Research Resources and Development, Kaohsiung Medical University, Kaohsiung 80708, Taiwan; ^3^ Department of Pathology, Kaohsiung Medical University Hospital, Kaohsiung Medical University, Kaohsiung 80708, Taiwan; ^4^ Department of General Surgery, Kaohsiung Medical University Hospital, Kaohsiung Medical University, Kaohsiung 80708, Taiwan; ^5^ Department of Obstetrics and Gynecology, Kaohsiung Medical University Hospital, Kaohsiung Medical University, Kaohsiung 80708, Taiwan; ^6^ Center for Stem Cell Research, Kaohsiung Medical University, Kaohsiung 80708, Taiwan; ^7^ Department of Cancer Biology, University of Cincinnati, College of Medicine, Cincinnati, Ohio 45267, USA

**Keywords:** miR-125a-5p, HDAC4, Tumorigenesis and breast cancer

## Abstract

Identifying stably expressed tumor markers that can be used easily to detect cancer is currently an important area of cancer research. By using miRNA microarray, we identified 20 differentially expressed miRNAs in serum samples of breast cancer patients. Expression of miR-125a-5p was relatively lower in patients with shorter survival compared to long-term survivors. In a cohort of breast cancer patients (*N* = 300), serum expression of miR-125a-5p was negatively and significantly correlated with tumor grade (*P* = 0.004), lymph-node status (*P* = 0.004), and tumor size (*P* < 0.001). Low miR-125a-5p expression was an independent prognostic marker (OR = 0.421; 95% CI = 0.184 to 0.961; *P* = 0.04) associated with poor survival rates (*P* = 0.0062). We show that miR-125a-5p directly inhibits expression of the HDAC4 gene, resulting in tumor suppression *in vitro* and *in vivo*. Together these results demonstrate that serum miR-125a-5p level in breast cancer may be a useful prognostic biomarker and offer a novel therapeutic avenue by targeting HDAC4 in breast cancer.

## INTRODUCTION

MicroRNAs (miRNAs) are short non-coding RNAs (19–25 nucleotides) that inhibit translation and induce mRNA degradation through binding to the 3′-untranslated region (UTR) of target mRNAs [1, 2]. A single miRNA can directly target many different mRNA sequences and, conversely, the same mRNA can harbor the target sites of several different miRNAs. Therefore, miRNAs and their mRNA targets constitute a regulatory network of cellular functions [3, 4]. Currently, about 20,000 human miRNAs have been recorded in the miRBase database (miRbase 18), and over 1500 different miRNAs have been reported in human cells [5]. In cancer cells, miRNAs are known to play critical roles in tumorigenesis by regulating cells growth, motility, angiogenesis, and apoptosis [6, 7]. In addition, miRNA is stably present in the serum of many cancer patients [8, 9], suggesting that serum miRNA can be explored as biomarkers for cancer diagnosis and prognosis [10–12]. In breast cancer, serum hsa-miR-21, miR-195, let-7a, and miR-10b have been reported as independent diagnostic and prognostic factors [10, 13, 14].

Histone deacetylases (HDACs) are the key enzymes regulating the acetylation status of both histone- and non-histone proteins [15]. On the chromatin, HDACs play important roles in regulating chromatin stability, transcription, and replication through their activities of compacting the chromatin, and preventing the recruitment of transcription factors and RNA polymerases. In addition, by altering the acetylation status of the substrate proteins, HDACs can indirectly modulate post-translational modifications such as phosphorylation, ubiquitylation, and sumoylation, thus navigating its influence through a wide spectrum of cellular functions [16, 17]. Early studies showed that HDACs influence the expression of numerous genes that are involved in cancer initiation and progression. Overexpression of HDACs promotes invasion, migration, angiogenesis, decreased adhesion, and decreased apoptosis in cancer cells [18]. Our previous study showed that HDAC6 was induced by endocrine disrupter chemicals and promoted tumorigenesis, epithelial-mesenchymal transition and angiogenesis of breast cancer [19–21]. Therefore, suppressing HDACs expression is an important direction in anti-cancer drug development [22].

In the current study, we analyze the association of miRNAs and breast cancer, and identify serum miRNAs as prognosis markers. This study leads to the discovery of a novel molecular mechanism in which the miRNA miR-125a-5p suppresses HDAC4 expression which can be exploited as a therapeutic approach of human breast cancer.

## RESULTS

### Association of circulating miR-125a-5p with clinicopathological characteristics and prognosis in human breast cancer patients

To investigate whether miRNAs are associated with survival in patients with breast cancer, we profiled miRNA expression in serum samples from five breast cancer patients (Table S1) who survived for less than 1 year after diagnosis (short-survival group) and five breast cancer patients who survived for more than 5 years after diagnosis (long-survival group) using an miRNA microarray (human 384 SeraMir qPCR Profiler array, System Biosciences). All patients had tumors positive for estrogen receptor, progesterone receptor, and HER2/ErbB2. Table S2 lists the identified miRNAs that were preferentially expressed in the short-survival group compared with the long-survival group. The results showed that miR-125a-5p expression was highly different in these two groups and showed relatively low expression levels in short-term survivors (Table 1).

**Table 1:** Differential expressions of miRNAs in Long Survival versus Short Survival Group were analyzed by miRNA microarray analysis

	Short Survival Group		Long Survival Group						
miRNA	Normalized	SD	Normalized	SD	Fold	Type	Function	Tissue type	Ref.
hsa-miR-125a-5p	0.05	0.08	1.95	0.02	0.03	down	low expression in cancer	Breast cancer	*Iorio et al., 2005*
hsa-miR-206	1.64	0.06	0.07	0.03	24.37	up	Unknown		
hsa-miR-146b-3p	0.2	0.13	3.93	0.06	0.05	down	Unknown		
hsa-miR-518a-3p	0.44	0.07	0.03	0.02	16.97	up	Unknown		
hsa-miR-193a-5p	1.91	0.05	0.13	0.06	14.88	up	Unknown		
hsa-miR-155	3.73	0.04	0.38	0.07	9.85	up	correlated with poor survival	Lung cancer	*Yanaihara et al., 2006*
hsa-miR-181c	1.2	0.08	10.31	0.09	0.12	down	regulates TNF-α expression	Hepatocellular	*Río et al., 2012*
hsa-miR-520c-3p	0.07	0.05	0.63	0.07	0.12	down	Unknown		
hsa-miR-30a	4.96	3.54	0.64	0.15	7.71	up	correlated with poor survival	Hepatocellular	*Budhu et al., 2008*
hsa-miR-181b	0.08	0.09	0.63	0.04	0.13	down	enhances drug sensitivity	Leukemia	*Zhu et al., 2012*
hsa-miR-503	24.03	0.07	3.15	0.06	7.63	up	regulates partial differentiation	Leukemia	*Forrest et al., 2010*
hsa-let-7b	0.02	0.05	0.12	0.02	0.15	down	regulates proliferation and apoptosis	Liver cancer	*Di Fazio et al., 2012*
hsa-let-7a	0.16	0.07	0.98	0.02	0.16	down	correlated with poor survival	Lung cancer	*Yanaihara et al., 2006*
hsa-miR-134	0.21	0.06	1.25	0.20	0.17	down	Unknown		
hsa-miR-486-5p	0.91	0.06	4.37	0.05	0.21	down	Unknown		
hsa-miR-21	0.45	0.04	0.09	0.03	4.77	up	correlated with poor survival	Lung carcinoma	*Gao et al., 2011*
hsa-miR-205	7.82	0.09	1.77	0.05	4.41	up	highly accurate marker for lung cancer	Lung carcinoma	*Lebanony et al., 2009*
hsa-miR-218	0.12	0.09	0.5	0.20	0.25	down	inhibits invasion and metastasis	Gastric cancer	*Tie et al., 2010*
hsa-miR-194	0.93	0.86	0.23	0.02	4.04	up	promote angiogenesis	Colon cancers	*Sundaram et al., 2011*
hsa-miR-151-3p	0.83	0.23	0.26	0.05	3.19	up	increases migration and invasion	Hepatocellular	*Ding et al., 2010*

Short Survival Group: survived for less than 1 year after diagnosis, case number = 5

Long Survival Group: survived for more than 5 years after diagnosis, case number = 5

Normalized: 2^^-Δct^, Fold: Short/Long

**Figure 1:**
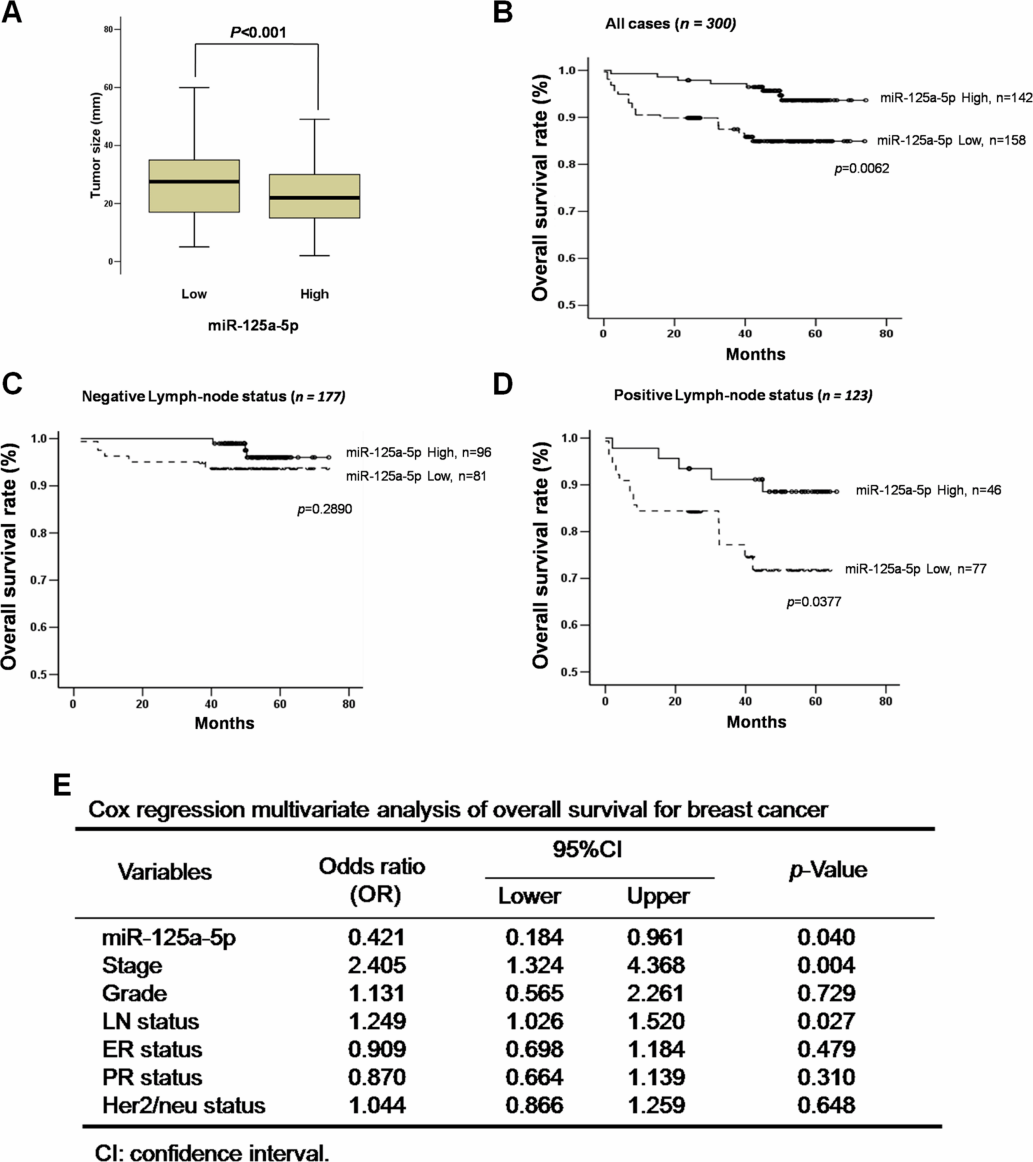
Low miR-125a-5p expression correlates with tumor size and poor survival in breast cancer patients **(A)** The average tumor size (mm in diameter) of the high miR-125a-5p level subgroup (*n* = 142) and the low miR-125a-5p level subgroup (*n* = 158). (B–D) Curves show the overall survival rates **(B)** and survival of patients with negative **(C)** or positive **(D)** lymph nodes in patients with high (solid line) versus low (dotted line) miR-125a-5p levels. **(E)** The multivariate Cox regression analysis of survival with the clinicopathological parameters and the miR-125a-5p expression.

To further understand the significance of miR-125a-5p expression in breast cancer patients, serum levels of miR-125a-5p were measured in the sera of 300 breast cancer patients by quantitative RT-PCR (qRT-PCR) and correlated with the clinicopathological parameters of these patients (Tables S3 and S4). We used median Ct miRNA expression level to define the high and low categories according to the previous reports [23, 24]. Patients were stratified into two groups based on the dichotomized scores (Table 2): high expression, miR-125a-5p expression > median (*n* = 142 patients); low expression, miR-125a-5p expression < or = median (*n* = 158 patients). The analysis showed that miR-125a-5p expression was inversely and significantly correlated with clinicopathological parameters including tumor grade (*p* = 0.004), lymph-node status (*p* = 0.004) (Table 2), and tumor size (*p* < 0.001) (Figure 1, A). The association of miR-125a-5p expression with overall patient survival and progression-free survival (PRS) based on lymph-node status was assessed by Kaplan–Meier analysis. Low miR-125a-5p expression was associated with lower survival rates (*p* = 0.0062) (Figure 1, B). Patients with positive lymph nodes (*n* = 123 patients) had the worst survival rate (*p* = 0.0377, Figure 1, D) compared to patients with negative lymph nodes (*n* = 177 patients, *p* = 0.2890, Figure 1, C) during a period of 80 months or longer of follow-up. In both groups, low level of miR125a-5p is associated with poor PRS.

**Table 2:** Relationship between miR-125a-5p expression level and clinicopathologic parameters of breast cancer

Variables	Number of cases *N* = 300	miR-125a-5p Low expression	High expression	*r*	*p*-Value
**Stage**				−0.104	0.071
I	121	58	63		
II	133	70	63		
III	46	30	16		
**Grade**				−0.167	0.004
I	128	54	74		
II	106	64	42		
III	66	40	26		
**Lymph-node status**				−0.166	0.004
Negative	177	81	96		
Positive	123	77	46		
**Estrogen receptor status**				−0.045	0.436
Negative	101	50	51		
Positive	199	108	91		
**Progesterone receptor status**				0.016	0.785
Negative	125	67	58		
Positive	175	91	84		
**Her2/Neu status**				0.108	0.061
Negative	202	114	88		
Positive	98	44	54		

Low expression: < or = Median, High expression: > Median*, P*: Two-sided χ2 test

Next, we performed multivariate Cox regression analysis with the clinicopathological parameters and miR-125a-5p expression. The level of miR-125a-5p expression (*p* = 0.04) and the stage (*p* = 0.004) were statistically significant predictors of breast cancer mortality (Figure 1, E). These data demonstrate that decreased miR-125a-5p was associated with breast cancer aggressiveness and may thus be a prognostic biomarker of breast cancer.

### miR-125a-5p overexpression decreases cancer cell growth and motility *in vitro*

**Figure 2:**
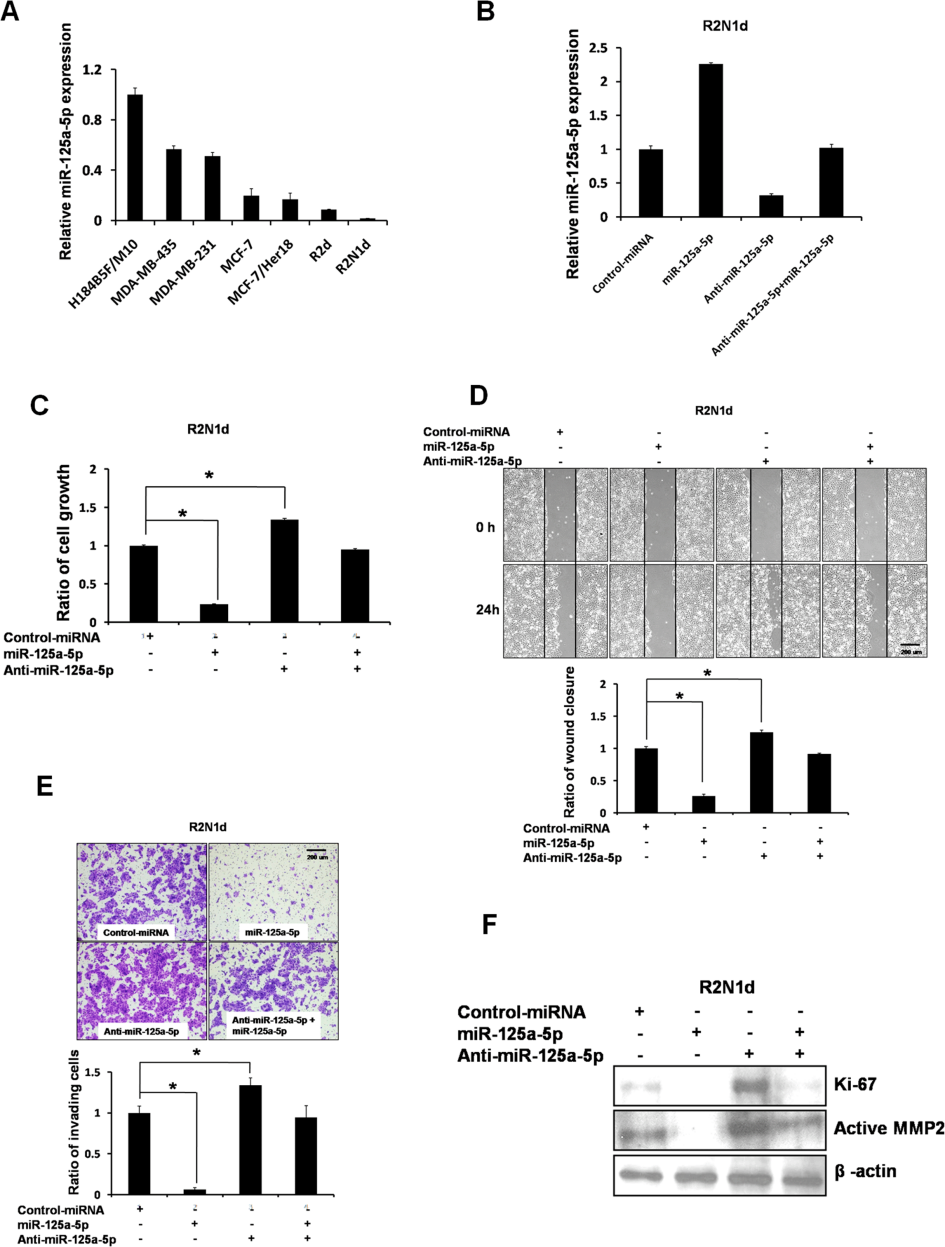
Expression and function of miR-125a-5p in breast cancer **(A)** miR-125a-5p expression in human normal breast cells (H184B5F/M10 cells) and breast cancer cell lines (MDA-MB-435, MDA-MB-231, MCF-7, MCF-7/Her18, R2d, R2N1d) was detected using q-PCR. **(B)** The R2N1d breast cancer cell line was transfected with control miRNA (5 μg), miR-125a-5p (5 μg), anti-miR-125a-5p (150 nmol/L), or anti-miR-125a-5p (150 nmol/L) + miR-125a-5p (5 μg), and miR-125a-5p expression was detected using q-PCR 48 hr post-transfection. (C–F) R2N1d cells were transfected as in (B). At the indicated times after transfection, the cells growth rate was evaluated by determining XTT assay **(C)**. The cells migration rate was evaluated with a wound-healing assay **(D)**. The cells invasion rate was evaluated using a transwell invasion chamber **(E)**. The proliferation marker, Ki-67 and the cells motility marker, MMP2 were evaluated with western blotting (F). Data are the means ± SD of three experiments. *P < 0.05 *vs*. untreated control; two-tailed Student's *t* test. Scale bar = 200 um.

We first analyzed using qRT-PCR the expression of miR-125a-5p in a cohort of breast cancer cell lines including MDA-MB-435, MDA-MB-231, MCF-7, its HER2/ErbB2-overexpressing derivative MCF-7/Her18, R2d, and R2N1d (Figure 2, A). Non-transformed breast epithelium cell line (H184B5F/M10) had the highest expression compared with malignant breast cancer cell lines. R2N1d, is a stem cell-like, highly malignant and metastatic cell line derived from human breast epithelial cells [25, 26], had the lowest miR-125a-5p expression in the group.

To examine the cellular function of miR-125a-5p, we overexpressed or depleted miR-125a-5p in R2N1d (Figure 2, B) and MDA-MB-231 (Figure S1A) cells. Down-regulation of miR-125a-5p promoted cells growth, migration, and invasion in R2N1d cells, which was abrogated by reconstitution of miR-125a-5p (Figure 2, C, D, E). Similar results were observed in MDA-MB-231 cells (Figure S1B, C, D). Consistently, down-regulation of miR-125a-5p in H184B5F/M10 induced its growth and migration activity (Figure S2A, B). To further confirm the biological function of miR-125a-5p in cells growth and migration, the levels of Ki-67 and MMP2 was examined with Western analysis. Overexpression of miR-125a-5p decreased Ki-67 and active MMP2 levels in both R2N1d (Figure 2, F) and MDA-MB-231 (Figure S1E) cells. These results together demonstrate an important role for miR-125a-5p in cells growth, migration and invasion of breast cancer cells.

### HDAC4 is a direct target of miR-125a-5p

**Figure 3:**
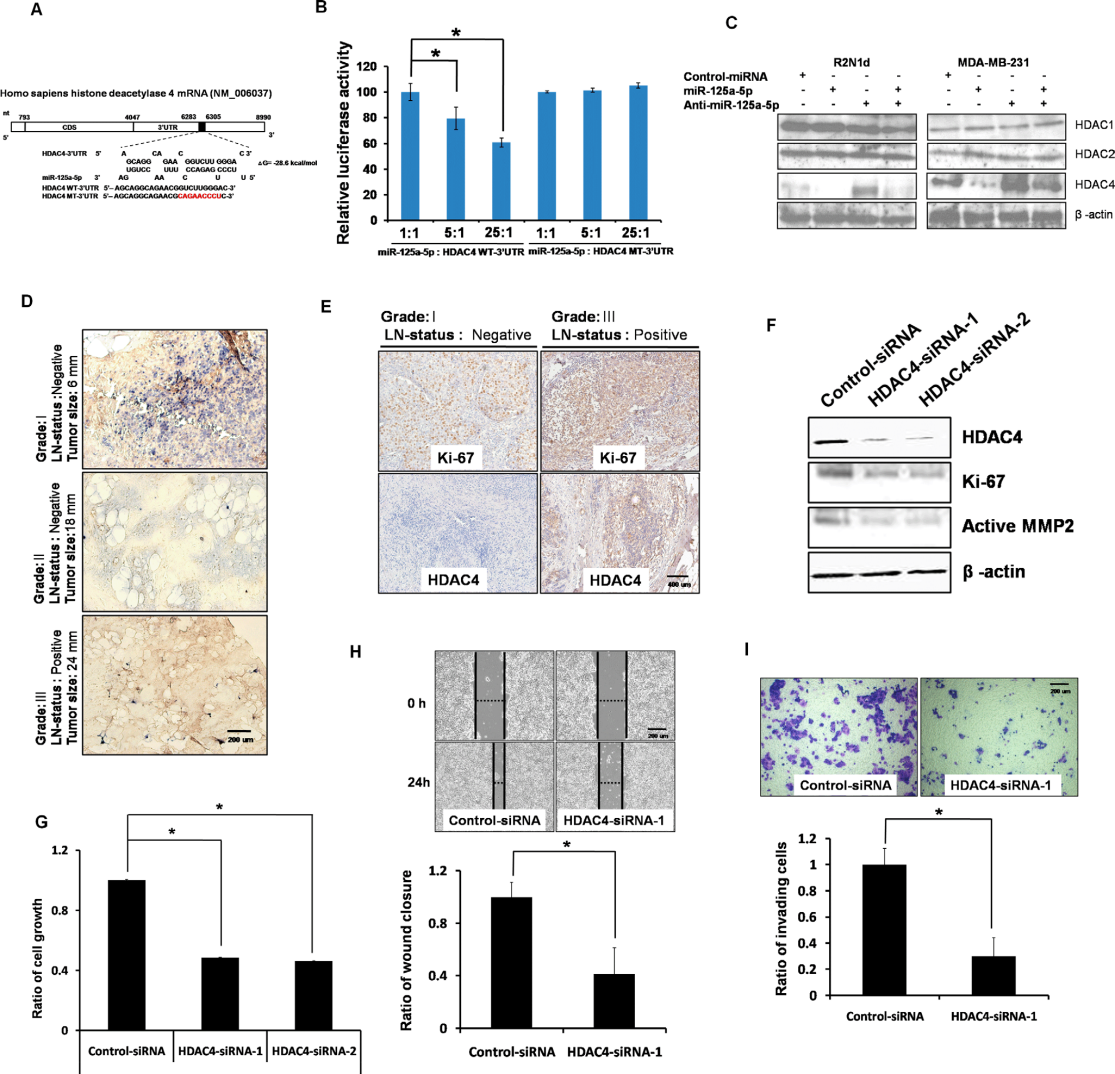
HDAC4 is a direct target of miR-125a-5p **(A)** HDAC4 gene 3′-UTRs contain one predicted miR-125a-5p binding site. **(B)** The 3′-UTRs of *HDAC4* were targets of miR-125a-5p. pGL3-HDAC luciferase constructs were prepared containing wild-type (WT-3′-UTR) or mutated (MT-3′-UTR) 3′-UTRs. 293T cells were cotransfected with HDAC4 3′-UTRs and a plasmid expressing miR-125a-5p at dose-dependent ratios. **(C)** R2N1d and MDA-MB-231 cells were transfected as in Figure 2B. HDAC4 protein expression was analyzed with western blotting 72 hr later. β-actin served as a loading control. **(D)** miR-125a-5p (blue) expression was analyzed with in situ hybridization in Grade I, II, and III breast cancer tissue, scale bar = 200 um. **(E)** Immunohistochemistry was performed to detect Ki-67 and HDAC4 in Grade I and III breast cancer tissue, scale bar = 400 um. **(F)** R2N1d breast cancer cells were transfected with control siRNA (5 μg), HDAC4 siRNA-1/2 (5 μg) and then protein expression of HDAC4, Ki-67 and MMP2 was detected using western blotting 72 hr post-transfection. (G–I) R2N1d cells were transfected as in **(F)**. At the indicated times after transfection, the cells growth rate was evaluated by determining XTT assay **(G)**. The cells migration rate was evaluated with a wound-healing assay, scale bar = 200 um (H). The cells invasion rate was evaluated in a transwell invasion chamber, scale bar = 200 um **(I)**. Data are the means ± SD of three experiments. *P < 0.05 *vs*. untreated control; two-tailed Student's *t* test.

To further investigate the target genes regulated by miR-125a-5p that may contribute to its biological function, several computational prediction methods and a publicly available algorithm were used to identify miR-125a-5p target genes in humans. Calculation using TargetScan (Human 5.1; [3]) indicated the most thermodynamically favorable interactions between the 5′-end of miR-125a-5p and the 3′-UTR of the *HDAC4* gene (Figure S3A). We therefore hypothesized that miR-125a-5p may suppress HDAC4 expression by directly binding to the target sites within the 3′-UTR of the *HDAC4* mRNA (Figure 3, A). To test this hypothesis, luciferase reporter vectors (PGL3) encoding wild-type (WT) and mutated (MT) 3′-UTRs of *HDAC4* was constructed and co-transfected with a miR-125a-5p plasmid into HEK-293T cells. We found that miR-125a-5p suppressed the luciferase reporter activity in a dose-dependent manner (Figure 3, B). In contrast, the mutant *HDAC4* construct, in which the miR-125a-5p target sequence was mutated, was unresponsive to miR-125a-5p. This result was confirmed by Western analysis showing that miR-125a-5p overexpression decreased HDAC4 protein levels *in vitro*, but not HDAC1 or HDAC2, which do not contain the targeting sequence of miR-125a-5p in their mRNA sequences (Figure 3, C). These data indicate that miR-125a-5p directly targets *HDAC4* in human breast cancer.

To examine the relationship between miR-125a-5p and HDAC4 in patients, *in situ* hybridization analysis was performed with 5′-digoxygenin–labeled locked nucleic acid (LNA) probes of miR-125a-5p on Grade I (lymph node–negative and tumor size = 6 mm), Grade II (lymph node–negative and tumor size = 18 mm), and Grade III (lymph node–positive and tumor size = 24 mm) breast cancer tissues, followed by immunohistochemistry with an anti-digoxygenin antibody. The results showed that miR-125a-5p expression was highest in Grade I compared with Grade II and Grade III tissues (Figure 3, D), which was consistent with previous experiments (Table 2). In contrast, HDAC4 expression as detected by immunohistochemical (IHC) staining using an anti-HDAC4 antibody was lowest in Grade I compared with Grade III tissues (Figure 3, E). Thus, miR-125a-5p is inversely correlated with HDAC4 in human breast tumors.

HDAC4 plays an important role in breast cancer growth and invasion. Depleting *HDAC4* by RNA interference down-regulated the levels of Ki-67 and active MMP2 (Figure 3, F). Depleting *HDAC4* also decreased cells growth, migration, and invasion in both R2N1d (Figure 3, G–I) and MDA-MB-231 (Figure S3, B-D) cells.

Previous studies have found that expression inhibition of a class I/II HDAC can lead to compensatory increase of other class I/II HDACs [27, 28]. To identify whether the down-regulation of HDAC4 impacted on other class II HDACs in human breast cancer, RNA expression of *HDAC5*, *HDAC7*, and *HDAC9* were examined in cells overexpressing miR-125a-5p or depleted for *HDAC4*. The results show that the expression of *HDAC4* and *HDAC5* was also decreased by overexpression of miR-125a-5p, while *HDAC7* and *HDAC9* were not affected. On the other hand, silencing *HDAC4* increased the expression of *HDAC5* and *HDAC7*, but *HDAC9* was not affected (Figure S4, A, B, C and D). Overall, these results suggest that miR-125a-5p blocks tumor development by targeting HDAC4.

### miR-125a-5p decreases growth, metastasis, and angiogenesis *in vivo*

**Figure 4:**
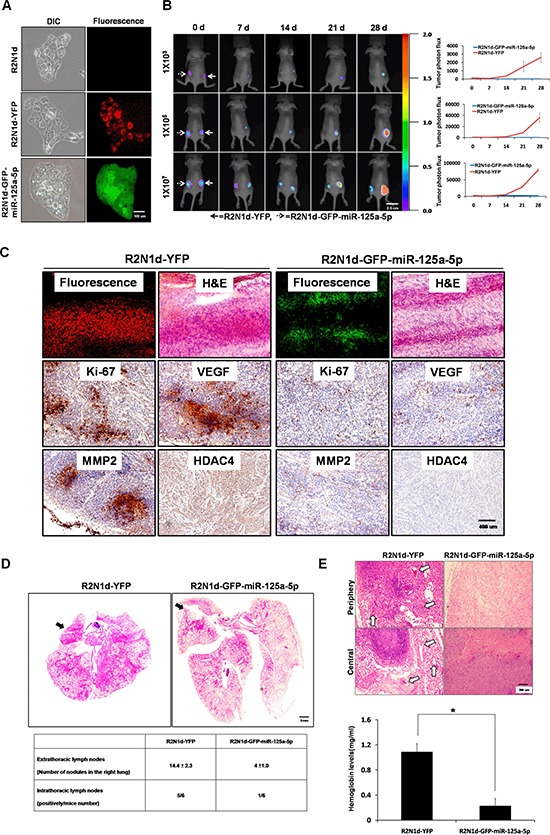
Tumor suppression functions of miR-125a-5p *in vivo* **(A)** R2N1d cells were infected with a YFP or GFP-miR-125a-5p plasmid, and the stable clones R2N1d-YFP and R2N1d-GFP-miR-125a-5p were obtained. GFP and YFP were detected with immunofluorescence, scale bar = 100 um. **(B)** R2N1d-YFP (solid arrows) and R2N1d-GFP-miR-125a-5p (dotted arrows) cells were injected into the right and left sides of immunodeficient SCID mice (*n* = 6 per group), respectively. The number of cells injected is shown to the left of each row. Left, bioluminescence imaging of the whole body of the mice was performed 0, 7, 14, 21, and 28 d after transplantation. Right, the mean fluorescence intensity was quantitated using MetaMorph software, scale bar = 2.5 cm. **(C)** The solid tumors derived from the nude mice were cut at a thickness of 5 μm. Fluorescence expression was analyzed with immunofluorescence, and H&E staining, and immunohistochemistry for Ki-67, VEGF, MMP2, and HDAC4 staining were performed, scale bar = 400 um. **(D)** R2N1d-YFP or R2N1d-GFP-miR-125a-5p cells were mixed with Matrigel and injected into the left lateral thorax of mice. Top, H&E staining of sections of the left and right lungs 7d after transplantation. Bottom, the extra- and intra-thoracic lymph nodes in the right lung were quantified with a dissecting microscope and pathologically confirmed by H&E staining. The arrow indicates the location of the injection, scale bar = 5 mm. **(E)** The cell were injected as in (B). Blood vessel formation was observed with H&E staining (top), and hemoglobin values were analyzed using Drabkin's reagent kit (bottom). The arrows show the location of the blood vessels, scale bar = 200 um.

To test the tumor suppression function of miR-125a-5p, R2N1d cells were infected with lentivirus-encoded pLKO.1-YFP or pLKO.1-GFP-miR-125a-5p plasmid and stable clones were generated by puromycin selection (referred to as R2N1d-YFP and R2N1d-GFP-miR-125a-5p). The R2N1d-GFP-miR-125a-5p cells, but not the R2N1d-YFP cells, exhibited membrane blebbing, a hallmark characteristic of apoptosis (Figure 4, A), which was confirmed by Annexin V staining showing that overexpression of miR-125a-5p increases the level of Annexin V in both R2N1d (Figure S5, A) and MDA-MB-231 cells (Figure S5, B).

Tumorigenesis was tested by subcutaneous inoculation of different numbers (1 × 10^3^, 1 × 10^5^ and 1 × 10^7^) of R2N1d-YFP and R2N1d-GFP-miR-125a-5p cells into nude mice (*n* = 6 per group). Whole-body bioluminescence detection was used to detect tumor growth on day 0, 7, 14, 21, and 28 after inoculation. R2N1d-GFP-miR-125a-5p cells yielded a significant lower mean fluorescence intensity compared with the R2N1d-YFP cells (Figure 4, B and Table S5)( 1 × 10^3^, R2N1d-YFP: 6/6, R2N1d-GFP-miR-125a-5p: 0/6 ; 1 × 10^5^, R2N1d-YFP: 6/6, R2N1d-GFP-miR-125a-5p: 0/6; 1 × 10^7^, R2N1d-YFP: 6/6, R2N1d-GFP-miR-125a-5p: 2/6). All tumor sections were positive for YFP or GFP fluorescence, confirming the origin of the tumors. IHC staining revealed that expression of Ki-67, vascular endothelial growth factor (VEGF), and MMP2 was low in R2N1d-GFP-miR-125a-5p tumors compared to R2N1d-YFP tumors (Figure 4, C). Consistent with the finding that miR-125a-5p suppresses HDAC4 expression *in vitro* (Figure 3), HDAC4 expression was lower in R2N1d-GFP-miR-125a-5p tumors than R2N1d-YFP tumors (Figure 4, C).

We then evaluated the role of miR-125a-5p during metastasis using a lung metastasis animal model in which R2N1d-YFP or R2N1d-GFP-miR-125a-5p cells were directly injected into the left lung through thorax of nude mice [29]. One week after injection, the lungs were removed, and the metastatic nodules of the right lung were counted and the tissue sections were stained by hematoxylin and eosin (H&E). We found that the tumor metastasis into the intra- and extra-thoracic lymph nodes of right lung was low for R2N1d-GFP-miR-125a-5p cells (intra, 1 nodules out of 6 injection; extra, number of nodules: 4.0 ± 1.0) compared to R2N1d-YFP cells (intra, 5 nodules out of 6 injectino; extra, number of nodules:14.4 ± 2.3) (Figure 4, D).

To assess angiogenic potential of these cells, matrigel plug assay was performed in nude mice. Tissue sections were stained with H&E, and hemoglobin in the plug was detected with Drabkin's reagent kit [21]. R2N1d-GFP-miR-125a-5p cells produced fewer functional blood vessels and lower hemoglobin levels compared with R2N1d-YFP cells (Figure 4, E). These data demonstrate that miR-125a-5p blocks the ability of breast cancer cells to grow, metastasize, and develop blood vessels *in vivo*.

### HDAC4 as a therapeutic target of miR-125a-5p

**Figure 5:**
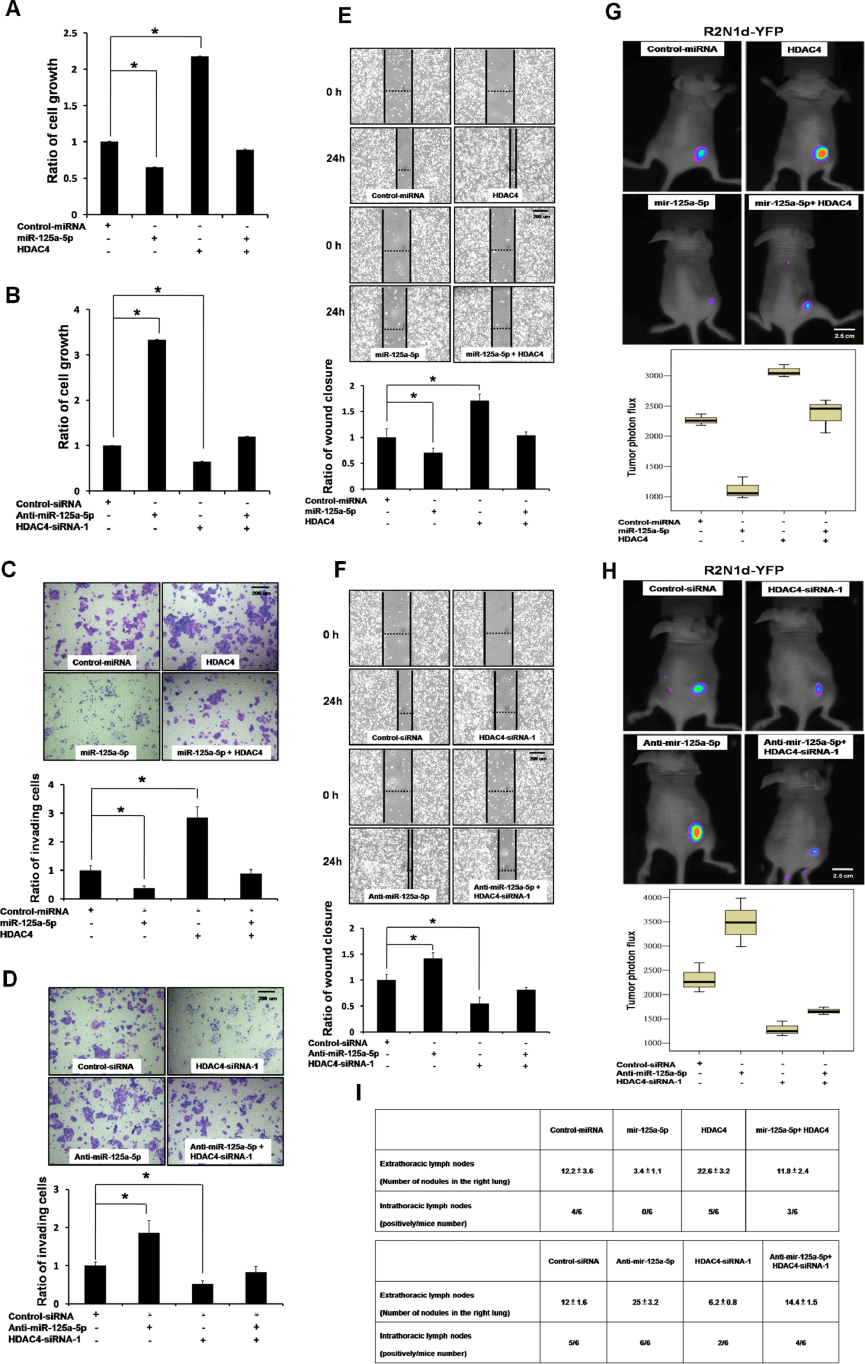
miR-125a-5p suppresses tumorigenesis through inhibition of HDAC4 R2N1d breast cancer cell line was transfected with 5 μg of each indicated plasmid or siRNAs, or anti-miR-125a-5p (150 nmol/L). Cell growth was evaluated by XTT assay **(A, B)**. Invasiveness was evaluated in a transwell invasion chamber **(C, D)**, and migration was evaluated with a wound-healing assay **(E, F)**. Data as the means ± SD were based on three independent experiments. *,*P* < 0.05 *vs*. untreated control as determined by two-tailed Student's *t* test. Scale bar = 200 um. **(G, H)** R2N1d-YFP cells were injected into the right sides of immunodeficient SCID mice (*n* = 6 per group). On day 7 after transplantation, mice were then treated with the indicated plasmids and reagents via intratumoral injection (10mg/kg; once a week injection). Tumor development was assessed by whole body bioluminescence imaging (top) of the 28 days after transplantation, and the mean fluorescence intensity was quantitated using the MetaMorph software (bottom). Scale bar = 2.5 cm. **(I)** R2N1d-YFP cells were mixed with matrigel and injected into the left lateral thorax of mice as in G and H. The extra- and intra-thoracic lymph nodes in the right lung were quantified with a dissecting microscope and pathologically confirmed by H&E staining.

These results together suggest a counteracting mechanism of miR-125a-5p and HDAC4 in tumor development which may be exploited as a therapeutic strategy of breast cancer. Indeed, overexpression of HDAC4 abolished miR-125a-5p-mediated inhibition in growth (Figure 5, A), invasion (Figure 5, C), migration (Figure 5, E), tumor growth (Figure 5, G) and metastasis (Figure 5, I) in R2N1d cells. Conversely, silencing of HDAC4 abolish anti-miR-125a-5p-induced growth (Figure 5, B), invasion (Figure 5, D) and migration (Figure 5, F) as well as tumor growth/metastasis (Figure 5, H, I). Importantly, depleting HDAC4 attenuated these tumor-enhancing activities, indicating that HDAC4 is a functional target of miR-125a-5p to suppress growth and tumor progression of breast cancer.

## DISCUSSION

In the current study we show that miR-125a-5p is negatively correlated with breast cancer progression and functions to inhibit tumor growth as well as metastasis by targeting HDAC4. Identifying tumor markers that can be easily detected to diagnose cancer is currently one of the most important areas of study in cancer research. Profiling of tumor miRNA has been reported in various cancers. Although numerous studies have demonstrated the involvement of miRNAs in breast cancer, the majority of these studies have focused on tissue samples. Despite that miRNA is stable and can be detected directly in serum [8], the miRNA population in serum has not been studied as assiduously. We have profiled serum miRNAs in ten breast cancer patients and correlated the results with patient survival. The top ten up-regulated miRNAs in the short-survival group (hsa-miR-206, hsa-miR-518a-3p, hsa-miR-193a-5p, hsa-miR-155, hsa-miR-30a, hsa-miR-503, hsa-miR-21, hsa-miR-205, hsa-miR-194, and hsa-miR-151-3p) were known to have potential oncogene activity in various cancers [30–36], whereas the top ten down-regulated miRNAs in the short-survival group (hsa-miR-125a-5p, hsa-miR-146b-3p, hsa-miR-181c, hsa-miR-520c-3p, hsa-miR-181b, hsa-miR-7b, hsa-miR-7a, hsa-miR-134, hsa-miR-486-5p and hsa-miR-218) were considered to be potential tumor suppressors [37–40]. Our results demonstrated that miRNAs in serum and tissue may have the same biological function and that detecting miRNAs in serum may be a feasible method for diagnosing breast cancer in patients. Our studies show that miR-125a-5p was particularly important as serum miR-125a-5p was associated with tumor stage and LN status. Low serum miR-125a-5p was strongly correlated with poor survival. Thus miR-125a-5p may serve as a new prognostic marker in clinical implication.

MiR-125a-5p was previously identified in an miRNA screen in normal human breast tissue and was shown to have decreased expression in human breast carcinoma [6]. Consistently, up-regulation of miR-125a-5p induces apoptosis through p53 activation in lung cancer [41]. In hepatocellular carcinoma, inhibiting miR-125a-5p increases mmp11 and vegfa protein expression, while restoring miR-125a-5p function inhibits proliferation and metastasis [42]. In addition, single-nucleotide polymorphism (snp) study has identified a G→T variant allele of miR-125a-5p. The t variant blocks the processing of pri-miRna to pre-mirna and is strongly associated with breast cancer risk [43]. A more recent report showed that miR-125a-5p plays an important role in inducing apoptosis through inhibiting the survival pathways mediated by aldehyde dehydrogenase 1a3 (aldh1a3) and myeloid leukemia–1 (mcl1) in colon cancer stem cells [44]. Our current studies provide a new ground by identification of the target genes of miR-125a-5p as a tumor suppressor and its application as a circulatory prognostic biomarker in advanced breast cancer.

Previous investigations have reported that HDACs play an important epigenetic role in regulating gene expression and cellular function in human cancer. Consistent with previous studies, our results suggest that cells respond to HDAC4 depletion by compensatory increase in the expression of HDAC5 and HDAC7 [27, 28]. Interestingly, miR-125a-5p not only targets HDAC4 down-regulation but also decreased the expression of HDAC5 in human breast cancer. Analysis of the 3′-UTR of HDAC5 revealed a miR-125a-5p target site (data not show). Therefore, it is likely that miR-125a-5p can bind to many different mRNA sequences and may require regulatory HDAC family member networks.

Early study found that overexpression of HDAC4 increases cell growth and tumor development by repressing p21^CIP1^ in cancer [45, 46]. HDAC4 interacts with hypoxia-inducible factor 1α (HIF-1a) and mediate angiogenesis in renal carcinoma cells [47]. HDAC4 has been proposed to contribute to cisplatin resistance of cancer cells by promoting STAT1 deacetylation [48]. Our results provide the first evidence that miR-125a-5p directly targets and blocks *HDAC4* expression by binding to the 3′-UTR of *HDAC4* mRNA. We further demonstrate the tumor suppression activity by miR-125a-5p expression or down-regulation of HDAC4 in human breast cancer.

In conclusion, our investigation has identified that miR-125a-5p in serum may be a convenient and reliable biomarker for prognosis in breast cancer and that miR-125a-5p plays an important role to suppress tumorigenesis by directly targeting HDAC4. These new findings are expected to lead to translational application in treatment and prognosis of human breast cancer.

## MATERIALS AND METHODS

### Cell lines and clinical specimens

The breast cancer cell lines H184B5F/M10, MDA-MB-435, MDA-MB-231, MCF-7, and MCF-7/Her18 were purchased from American Type Culture Collection (ATCC). The cancer stem cell lines R2d and R2N1d were a kind gift from Prof. C.-C. Chang (Michigan State University, East Lansing, MI) and were cultured as described [25]. Human breast cancer specimens were collected from the Kaohsiung Medical University Hospital. The protocol was approved by the Kaohsiung Medical University Institutional Review Board Committee (KMUH-IRB-990319, 20110239).

### Luciferase assay

HEK-293T cells were cotransfected with PGL3-control-3′-UTR (Promega, Madison, WI), PGL3-HDAC4-WT-3′-UTR, or PGL3-HDAC4-MT-3′-UTR, and the indicated amounts of miR-125a-5p using TurboFect Transfection Reagent (Fermentas, Vilnius, Lithuania). Cells were cultured for 24 hr after transfection, and activity was measured with the Dual-Glo Luciferase Assay (Promega, Madison, WI) according to the manufacturer's protocol. The sequences of the 3′-UTR constructs are shown in Figure 3a.

### *In vivo* experiments

Animal studies were performed according to protocols approved by the Kaohsiung Medical University Institutional Animal Care and Use Committee (IACUC Approval No: 101036). Female mice (BALB/cAnN. Cg-*Foxn1nu*/Crl-Narl, 4 to 6 weeks old) were obtained from the National Laboratory Animal Center (Taipei, Taiwan). R2N1d cells were infected with viruses carrying pLKO.1-YFP (National RNAi Core Facility, Academia Sinica, Taipei, Taiwan) or pLKO.1-GFP-miR-125a-5p plasmids according to the RNAi Core Facility's protocol. For the xenograft model, cells stably expressing YFP or GFP-miR-125a-5p were injected subcutaneously into the flanks of nude mice, and the fluorescent density was measured 7, 14, 21, and 28 d after injection using an Ultra Sensitive Molecular Imaging System (Berthold Technologies, Bad Wildbad, Germany). For the metastasis model, R2N1d-YFP or R2N1d-GFP-miR-125a-5p cells were mixed with Matrigel (1:1, BD Biosciences, San Jose, CA) and injected into the left lateral thorax of nude mice as described [29]. The extra- and intra-thoracic lymph nodes in the right lung were quantified with a dissecting microscope and pathologically confirmed by H&E staining. For the matrigel plug angiogenesis model, the cells were resuspended and mixed with Matrigel (1:1) and then injected into the flanks of nude mice as described [21]. Fifteen days after implantation, blood vessel formation was determined with H&E staining, and hemoglobin values were analyzed using Drabkin's reagent kit (Sigma, St Louis, MO).

### miRNA isolation and quantitative Real-Time PCR

Total RNA was extracted from serum using the MasterPure Complete DNA & RNA Purification kit (EPICENTRE Biotechnologies, Madison, WI) according to the manufacturer's guidelines. miRNA was amplified using the corresponding reverse transcription primer and the TaqMan MicroRNA Reverse Transcription kit (Applied Biosystems, Foster City, CA). *miR-16* was used for normalization of miRNA amounts in serum [9, 49], and the 2^^−ΔΔ^Ct method was used to determine the relative expression.

### In situ hybridization and immunohistochemistry

For in situ hybridization, miR-125a-5p in tissues sections was detected using a 5′-digoxygenin–labeled *miR-125a-5p* miRCURY™ LNA detection probe (Exiqon, Vedbaek, Denmark) and an IsHyb In Situ Hybridization kit (BioChain, Hayward, CA) according to the manufacturer's protocol. The probe sequence was 5′-TCACAGGTTAAAGGGTCTCAGGGA-3′. For immunohistochemistry, 5-μm thick sections were deparaffinized with xylene and dehydrated using ethanol. Immunohistochemistry staining was performed with a Dako LSAB kit (Dako, Carpinteria, CA) according to the manufacturer's protocol. The nuclei were counterstained with hematoxylin. The following antibodies were used for immunohistochemistry: HDAC Family Antibody Set (1:1000, Biovision, Mountain View, CA), anti–Ki-67 (1:1000, Sigma), anti-VEGF (1:500, Santa Cruz Biotech, Santa Cruz, CA), anti-MMP2 (1:1000, Cell Signaling, Beverly, MA).

### Immunoblot analysis

Cell lysates were prepared with the M-PER mammalian protein extraction reagent (Thermo Scientific, Franklin, MA) and stored at –20°C until use. For immunoblot analysis, cell lysates were resolved on SDS/PAGE gels and blotted onto polyvinylidene difluoride membranes (Millipore, Bedford, MA). Membranes were probed with antibodies at 4°C for 24 hr and developed with the ECL plus Western Blotting kit (Millipore). The following antibodies were used for immunoblotting: HDAC Family Antibody Set (1:1000, Biovision), anti–Ki-67 (1:1000, Sigma), anti-MMP2 (1:1000, Cell Signaling).

### Transfection and plasmids, siRNA, and shRNA

Cells were seeded into a 6-well plate, incubated for 24 hr, and then transfected with plasmid or RNA using TurboFect Transfection Reagent (Fermentas, Hanover, MD) according to the manufacturer's guidelines. The following plasmid and RNAs were used: pLKO. TRC-miR-125a-5p: 5′-UCCCUGAGACCCUUUAACCUGUG 3′-ends, pLKO.1-HDAC4 shRNA-1: 5′-CGACTCATCTTG TAGCTTATT 3′-ends, pLKO.1-HDAC4 shRNA-2: 5′-GAATCTGAACCACTGCATTTC 3′-ends.

### Cell growth, invasion, and wound-healing assays

Cell growth was assessed using the 3′-(1-(phenylaminocarbony)-3,4-tetrazolium)-*bis* - (4-methoxy-6-nitro)-benzene sulfonic acid hydrate (XTT) solution (Sigma), and absorbance at 490 nm (650 nm reference for the XTT solution) was measured in an ELISA reader (Multiskan EX; Labsystems, Vantaa, Finland). For the wound healing assay, the cells were cultured for 24 h (90% confluency) and scratched with a micropipette tip in a six-well plate. 24 h later, the wound width was captured by light microscope (Olympus, Tokyo, Japan) and wound closure was measured at three defined positions along the scratch. The invasiveness of cells was evaluated by a Cell Invasion Assay kit according to the manufacturer's instructions (Chemicon, Temecula, CA). Briefly, the invading cells on the lower surface of the membrane were stained with crystal violet (Sigma) and photographs were captured by an Olympus microscope [19, 20].

### Immunofluorescence and apoptosis assay

The YFP- or GFP-miR-125a-5p–expressing cells and tissues sections were fixed for 20 min in 4% paraformaldehyde. Cells were observed with an IX-71 microscope and analyzed with DP2-BSW software (Olympus, Tokyo, Japan). For apoptosis assay, the cells were analyzed with annexin V-FITC apoptosis kit (BD Biosciences Pharmingen, San Diego, CA). The cells were collected and stained with propidium iodide (PI) and Annexin V. After 30 min, the samples were analyzed by flow cytometry.

### Statistical analysis

Two-sided Student's t test was performed for comparisons between short and long survival group in microarray analysis. Two-sided χ2 test was determined for comparisons between miR-125a-5p high-expression and low-expression groups for tumor stage, tumor grade, tumor size, lymph-nodes status, estrogen receptor status, progesterone receptor status, and HER2/Neu status. Survival curves and 95% confidence intervals (CI) were evaluated by Kaplan-Meier estimates and multivariable Cox proportional hazards regression models, respectively. All of the statistical analyses were performed by the SPSS 12.0 (Chicago, IL) statistical software.

## SUPPLEMENTARY FIGURES AND TABLES



## References

[1] Ambros V, Lee RC (2004). Identification of microRNAs and other tiny noncoding RNAs by cDNA cloning. Methods Mol Biol.

[2] Lai EC (2004). Predicting and validating microRNA targets. Genome biology.

[3] Lewis BP, Shih IH, Jones-Rhoades MW, Bartel DP, Burge CB (2003). Prediction of mammalian microRNA targets. Cell.

[4] Volinia S, Galasso M, Sana ME, Wise TF, Palatini J, Huebner K, Croce CM (2012). Breast cancer signatures for invasiveness and prognosis defined by deep sequencing of microRNA. Proceedings of the National Academy of Sciences of the United States of America.

[5] Kozomara A, Griffiths-Jones S (2011). miRBase: integrating microRNA annotation and deep-sequencing data. Nucleic acids research.

[6] Iorio MV, Ferracin M, Liu CG, Veronese A, Spizzo R, Sabbioni S, Magri E, Pedriali M, Fabbri M, Campiglio M, Menard S, Palazzo JP, Rosenberg A, Musiani P, Volinia S, Nenci I (2005). MicroRNA gene expression deregulation in human breast cancer. Cancer research.

[7] Shen J, Xia W, Khotskaya YB, Huo L, Nakanishi K, Lim SO, Du Y, Wang Y, Chang WC, Chen CH, Hsu JL, Wu Y, Lam YC, James BP, Liu X, Liu CG (2013). EGFR modulates microRNA maturation in response to hypoxia through phosphorylation of AGO2. Nature.

[8] Chen X, Ba Y, Ma L, Cai X, Yin Y, Wang K, Guo J, Zhang Y, Chen J, Guo X, Li Q, Li X, Wang W, Wang J, Jiang X, Xiang Y (2008). Characterization of microRNAs in serum: a novel class of biomarkers for diagnosis of cancer and other diseases. Cell research.

[9] Mitchell PS, Parkin RK, Kroh EM, Fritz BR, Wyman SK, Pogosova-Agadjanyan EL, Peterson A, Noteboom J, O'Briant KC, Allen A, Lin DW, Urban N, Drescher CW, Knudsen BS, Stirewalt DL, Gentleman R (2008). Circulating microRNAs as stable blood-based markers for cancer detection. Proceedings of the National Academy of Sciences of the United States of America.

[10] Wittmann J, Jack HM (2010). Serum microRNAs as powerful cancer biomarkers. Biochimica et biophysica acta.

[11] Wang Y, Gu J, Roth JA, Hildebrandt MA, Lippman SM, Ye Y, Minna JD, Wu X (2013). Pathway-based serum microRNA profiling and survival in patients with advanced stage non-small cell lung cancer. Cancer research.

[12] Hsu CY, Hsieh TH, Tsai CF, Tsai HP, Chen HS, Chang Y, Chuang HY, Lee JN, Hsu YL, Tsai EM (2013). miRNA-199a-5p regulates VEGFA in endometrial mesenchymal stem cells and contributes to the pathogenesis of endometriosis. The Journal of pathology.

[13] Asaga S, Kuo C, Nguyen T, Terpenning M, Giuliano AE, Hoon DS (2011). Direct serum assay for microRNA-21 concentrations in early and advanced breast cancer. Clinical chemistry.

[14] Ma L, Teruya-Feldstein J, Weinberg RA (2007). Tumour invasion and metastasis initiated by microRNA-10b in breast cancer. Nature.

[15] Glozak MA, Sengupta N, Zhang X, Seto E (2005). Acetylation and deacetylation of non-histone proteins. Gene.

[16] Brandl A, Heinzel T, Kramer OH (2009). Histone deacetylases: salesmen and customers in the post-translational modification market. Biology of the cell.

[17] Xu WS, Parmigiani RB, Marks PA (2007). Histone deacetylase inhibitors: molecular mechanisms of action. Oncogene.

[18] Glozak MA, Seto E (2007). Histone deacetylases and cancer. Oncogene.

[19] Hsieh TH, Tsai CF, Hsu CY, Kuo PL, Lee JN, Chai CY, Wang SC, Tsai EM (2012). Phthalates induce proliferation and invasiveness of estrogen receptor-negative breast cancer through the AhR/HDAC6/c-Myc signaling pathway. FASEB journal.

[20] Hsieh TH, Tsai CF, Hsu CY, Kuo PL, Lee JN, Chai CY, Hou MF, Chang CC, Long CY, Ko YC, Tsai EM (2012). Phthalates stimulate the epithelial to mesenchymal transition through an HDAC6-dependent mechanism in human breast epithelial stem cells. Toxicological sciences.

[21] Hsieh TH, Tsai CF, Hsu CY, Kuo PL, Hsi E, Suen JL, Hung CH, Lee JN, Chai CY, Wang SC, Tsai EM (2012). n-Butyl benzyl phthalate promotes breast cancer progression by inducing expression of lymphoid enhancer factor 1. PloS one.

[22] Mitsiades CS, Mitsiades NS, McMullan CJ, Poulaki V, Shringarpure R, Hideshima T, Akiyama M, Chauhan D, Munshi N, Gu X, Bailey C, Joseph M, Libermann TA, Richon VM, Marks PA, Anderson KC (2004). Transcriptional signature of histone deacetylase inhibition in multiple myeloma: biological and clinical implications. Proceedings of the National Academy of Sciences of the United States of America.

[23] Li A, Omura N, Hong SM, Vincent A, Walter K, Griffith M, Borges M, Goggins M (2010). Pancreatic cancers epigenetically silence SIP1 and hypomethylate and overexpress miR-200a/200b in association with elevated circulating miR-200a and miR-200b levels. Cancer research.

[24] Heegaard NH, Schetter AJ, Welsh JA, Yoneda M, Bowman ED, Harris CC (2012). Circulating micro-RNA expression profiles in early stage nonsmall cell lung cancer. International journal of cancer.

[25] Kao CY, Nomata K, Oakley CS, Welsch CW, Chang CC (1995). Two types of normal human breast epithelial cells derived from reduction mammoplasty: phenotypic characterization and response to SV40 transfection. Carcinogenesis.

[26] Wang KH, Kao AP, Chang CC, Lee JN, Hou MF, Long CY, Chen HS, Tsai EM (2010). Increasing CD44+/CD24(−) tumor stem cells, and upregulation of COX-2 and HDAC6, as major functions of HER2 in breast tumorigenesis. Molecular cancer.

[27] Mihaylova MM, Vasquez DS, Ravnskjaer K, Denechaud PD, Yu RT, Alvarez JG, Downes M, Evans RM, Montminy M, Shaw RJ (2011). Class IIa histone deacetylases are hormone-activated regulators of FOXO and mammalian glucose homeostasis. Cell.

[28] Clocchiatti A, Di Giorgio E, Ingrao S, Meyer-Almes FJ, Tripodo C, Brancolini C (2013). Class IIa HDACs repressive activities on MEF2-depedent transcription are associated with poor prognosis of ER(+) breast tumors. FASEB journal.

[29] Onn A, Isobe T, Itasaka S, Wu W, O'Reilly MS, Ki Hong W, Fidler IJ, Herbst RS (2003). Development of an orthotopic model to study the biology and therapy of primary human lung cancer in nude mice. Clinical cancer research.

[30] Yanaihara N, Caplen N, Bowman E, Seike M, Kumamoto K, Yi M, Stephens RM, Okamoto A, Yokota J, Tanaka T, Calin GA, Liu CG, Croce CM, Harris CC (2006). Unique microRNA molecular profiles in lung cancer diagnosis and prognosis. Cancer cell.

[31] Budhu A, Jia HL, Forgues M, Liu CG, Goldstein D, Lam A, Zanetti KA, Ye QH, Qin LX, Croce CM, Tang ZY, Wang XW (2008). Identification of metastasis-related microRNAs in hepatocellular carcinoma. Hepatology.

[32] Forrest AR, Kanamori-Katayama M, Tomaru Y, Lassmann T, Ninomiya N, Takahashi Y, de Hoon MJ, Kubosaki A, Kaiho A, Suzuki M, Yasuda J, Kawai J, Hayashizaki Y, Hume DA, Suzuki H (2010). Induction of microRNAs, mir-155, mir-222, mir-424 and mir-503, promotes monocytic differentiation through combinatorial regulation. Leukemia.

[33] Gao W, Shen H, Liu L, Xu J, Shu Y (2011). MiR-21 overexpression in human primary squamous cell lung carcinoma is associated with poor patient prognosis. Journal of cancer research and clinical oncology.

[34] Lebanony D, Benjamin H, Gilad S, Ezagouri M, Dov A, Ashkenazi K, Gefen N, Izraeli S, Rechavi G, Pass H, Nonaka D, Li J, Spector Y, Rosenfeld N, Chajut A, Cohen D (2009). Diagnostic assay based on hsa-miR-205 expression distinguishes squamous from nonsquamous non-small-cell lung carcinoma. Journal of clinical oncology.

[35] Sundaram P, Hultine S, Smith LM, Dews M, Fox JL, Biyashev D, Schelter JM, Huang Q, Cleary MA, Volpert OV, Thomas-Tikhonenko A (2011). p53-responsive miR-194 inhibits thrombospondin-1 and promotes angiogenesis in colon cancers. Cancer research.

[36] Ding J, Huang S, Wu S, Zhao Y, Liang L, Yan M, Ge C, Yao J, Chen T, Wan D, Wang H, Gu J, Yao M, Li J, Tu H, He X (2010). Gain of miR-151 on chromosome 8q24.3 facilitates tumour cell migration and spreading through downregulating RhoGDIA. Nature cell biology.

[37] Rio P, Agirre X, Garate L, Banos R, Alvarez L, San Jose-Eneriz E, Badell I, Casado JA, Garin M, Prosper F, Bueren JA (2012). Down-regulated expression of hsa-miR-181c in Fanconi anemia patients: implications in TNFalpha regulation and proliferation of hematopoietic progenitor cells. Blood.

[38] Zhu DX, Zhu W, Fang C, Fan L, Zou ZJ, Wang YH, Liu P, Hong M, Miao KR, Xu W, Li JY (2012). miR-181a/b significantly enhances drug sensitivity in chronic lymphocytic leukemia cells via targeting multiple anti-apoptosis genes. Carcinogenesis.

[39] Tie J, Pan Y, Zhao L, Wu K, Liu J, Sun S, Guo X, Wang B, Gang Y, Zhang Y, Li Q, Qiao T, Zhao Q, Nie Y, Fan D (2010). MiR-218 inhibits invasion and metastasis of gastric cancer by targeting the Robo1 receptor. PLoS genetics.

[40] Di Fazio P, Montalbano R, Neureiter D, Alinger B, Schmidt A, Merkel AL, Quint K, Ocker M (2012). Downregulation of HMGA2 by the pan-deacetylase inhibitor panobinostat is dependent on hsa-let-7b expression in liver cancer cell lines. Experimental cell research.

[41] Jiang L, Huang Q, Chang J, Wang E, Qiu X (2011). MicroRNA HSA-miR-125a-5p induces apoptosis by activating p53 in lung cancer cells. Experimental lung research.

[42] Bi Q, Tang S, Xia L, Du R, Fan R, Gao L, Jin J, Liang S, Chen Z, Xu G, Nie Y, Wu K, Liu J, Shi Y, Ding J, Fan D (2012). Ectopic expression of MiR-125a inhibits the proliferation and metastasis of hepatocellular carcinoma by targeting MMP11 and VEGF. PloS one.

[43] Peterlongo P, Caleca L, Cattaneo E, Ravagnani F, Bianchi T, Galastri L, Bernard L, Ficarazzi F, Dall'olio V, Marme F, Langheinz A, Sohn C, Burwinkel B, Giles GG, Baglietto L, Severi G (2011). The rs12975333 variant in the miR-125a and breast cancer risk in Germany, Italy, Australia and Spain. Journal of medical genetics.

[44] Chen J, Chen Y, Chen Z (2013). miR-125a/b Regulates the Activation of Cancer Stem Cells in Paclitaxel-resistant Colon Cancer. Cancer investigation.

[45] Wilson AJ, Byun DS, Nasser S, Murray LB, Ayyanar K, Arango D, Figueroa M, Melnick A, Kao GD, Augenlicht LH, Mariadason JM (2008). HDAC4 promotes growth of colon cancer cells via repression of p21. Molecular biology of the cell.

[46] Mottet D, Pirotte S, Lamour V, Hagedorn M, Javerzat S, Bikfalvi A, Bellahcene A, Verdin E, Castronovo V (2009). HDAC4 represses p21(WAF1/Cip1) expression in human cancer cells through a Sp1-dependent, p53-independent mechanism. Oncogene.

[47] Qian DZ, Kachhap SK, Collis SJ, Verheul HM, Carducci MA, Atadja P, Pili R (2006). Class II histone deacetylases are associated with VHL-independent regulation of hypoxia-inducible factor 1 alpha. Cancer research.

[48] Stronach EA, Alfraidi A, Rama N, Datler C, Studd JB, Agarwal R, Guney TG, Gourley C, Hennessy BT, Mills GB, Mai A, Brown R, Dina R, Gabra H (2011). HDAC4-regulated STAT1 activation mediates platinum resistance in ovarian cancer. Cancer research.

[49] Hoffman AE, Zheng T, Yi C, Leaderer D, Weidhaas J, Slack F, Zhang Y, Paranjape T, Zhu Y (2009). microRNA miR-196a-2 and breast cancer: a genetic and epigenetic association study and functional analysis. Cancer research.

