# Supplementary material for: miR-125a-5p is a prognostic biomarker that targets HDAC4 to suppress breast tumorigenesis
Source: Oncotarget. 2014 Nov 28;6(1):494–509. doi: 10.18632/oncotarget.2674 (PMC4381610; doi:10.18632/oncotarget.2674)
Supplement: Supplementary file 1 [file oncotarget-06-494-s001.pdf]

SUPPLEMENTARY FIGURES AND TABLES

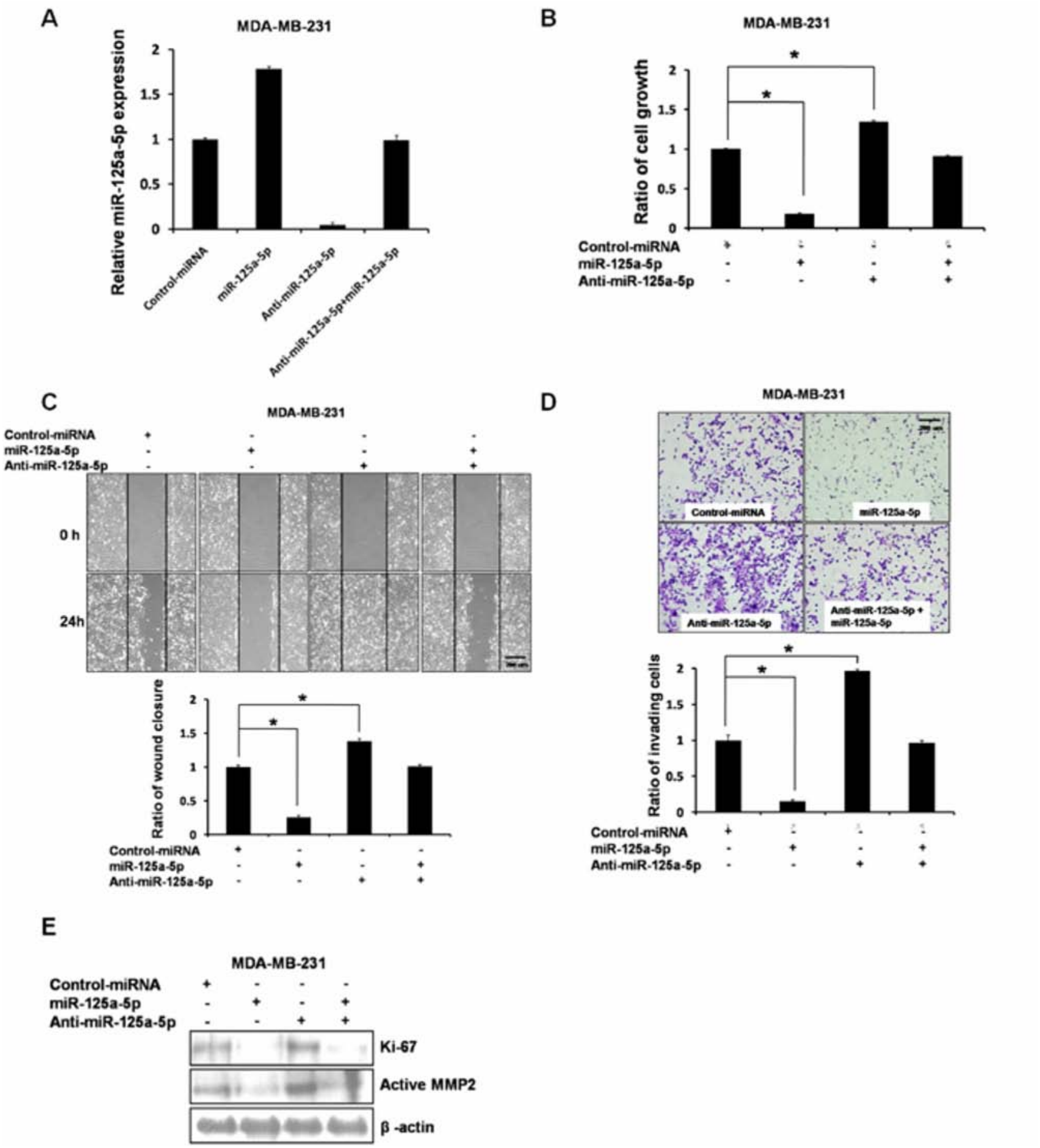

Supplementary Figure S1: miR-125a-5p decreases growth and motility in MDA-MB-231 breast cancer cells. (A) MDA-MB-231 breast cancer cells were transfected with control miRNA (5 μg), miR-125a-5p (5 μg), anti-miR-125a-5p (150 nmol/L), or anti-miR-125a-5p (150 nmol/L) + miR-125a-5p (5 μg), and miR-125a-5p expression was detected with qRT-PCR 48 hr after transfection. **(B–E)** MDA-MB-231 cells were transfected as in (A). At the indicated times after transfection, cell growth was evaluated by counting the cell number (B). Cell migration rate was evaluated with wound-healing assay (C). Cell invasion was evaluated with a transwell invasion chamber (D). At the indicated post-transfection times, the proliferation marker Ki-67 and the motility marker MMP2 were evaluated with Western blotting (E). Data are the means ± SD of three experiments. *$P < 0.05$ *vs*. untreated control determined by two-tailed Student's *t* test. Scale bar = 200 um.

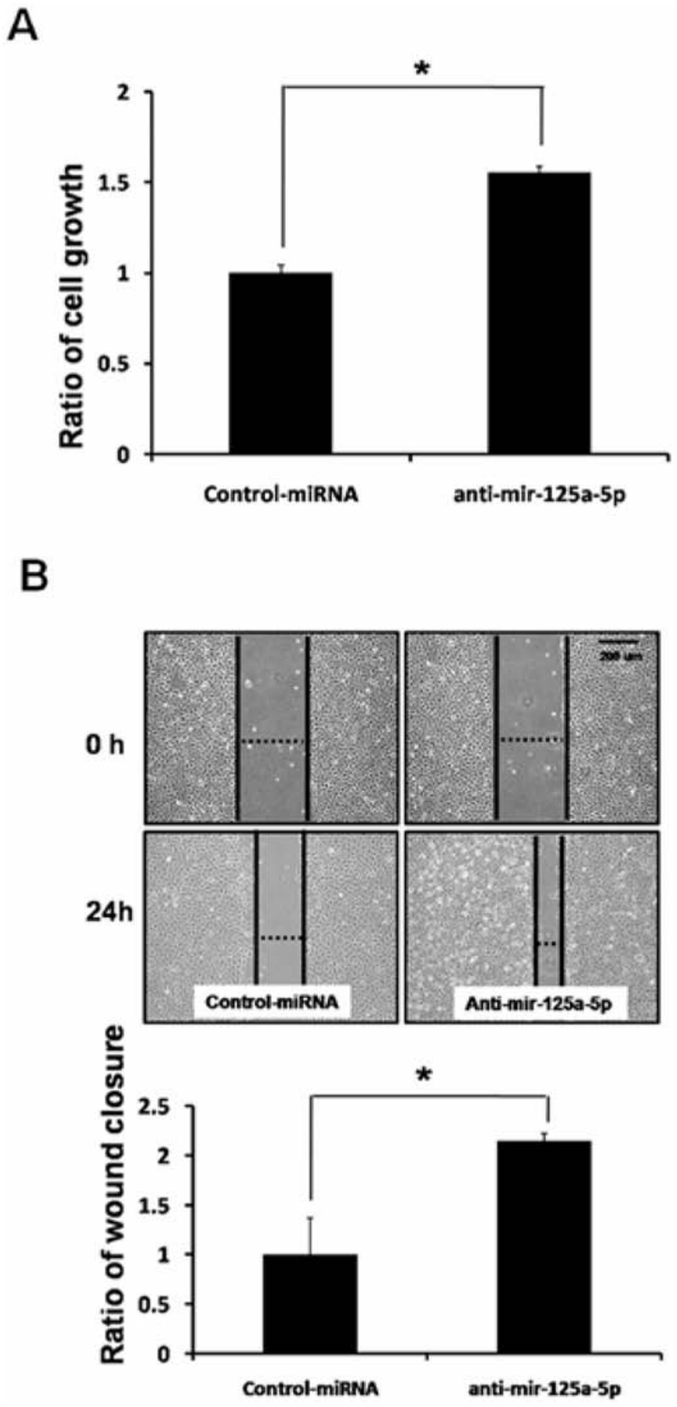

Supplementary Figure S2: miR-125a-5p inhibition promoted growth and migration of H184B5F/M10 cells. H184B5F/M10 cells were transfected with control-miRNA (5 µg) or anti-miR-125a-5p (150 nmol/L). At the indicated times after transfection, the cell growth was determined by XTT assay **(A)**. Cell migration was evaluated with the wound-healing assay **(B)**. Data are the means ± SD of three experiments. **P* < 0.05 *vs*. untreated control determined by two-tailed Student's *t* test. Scale bar = 200 um.

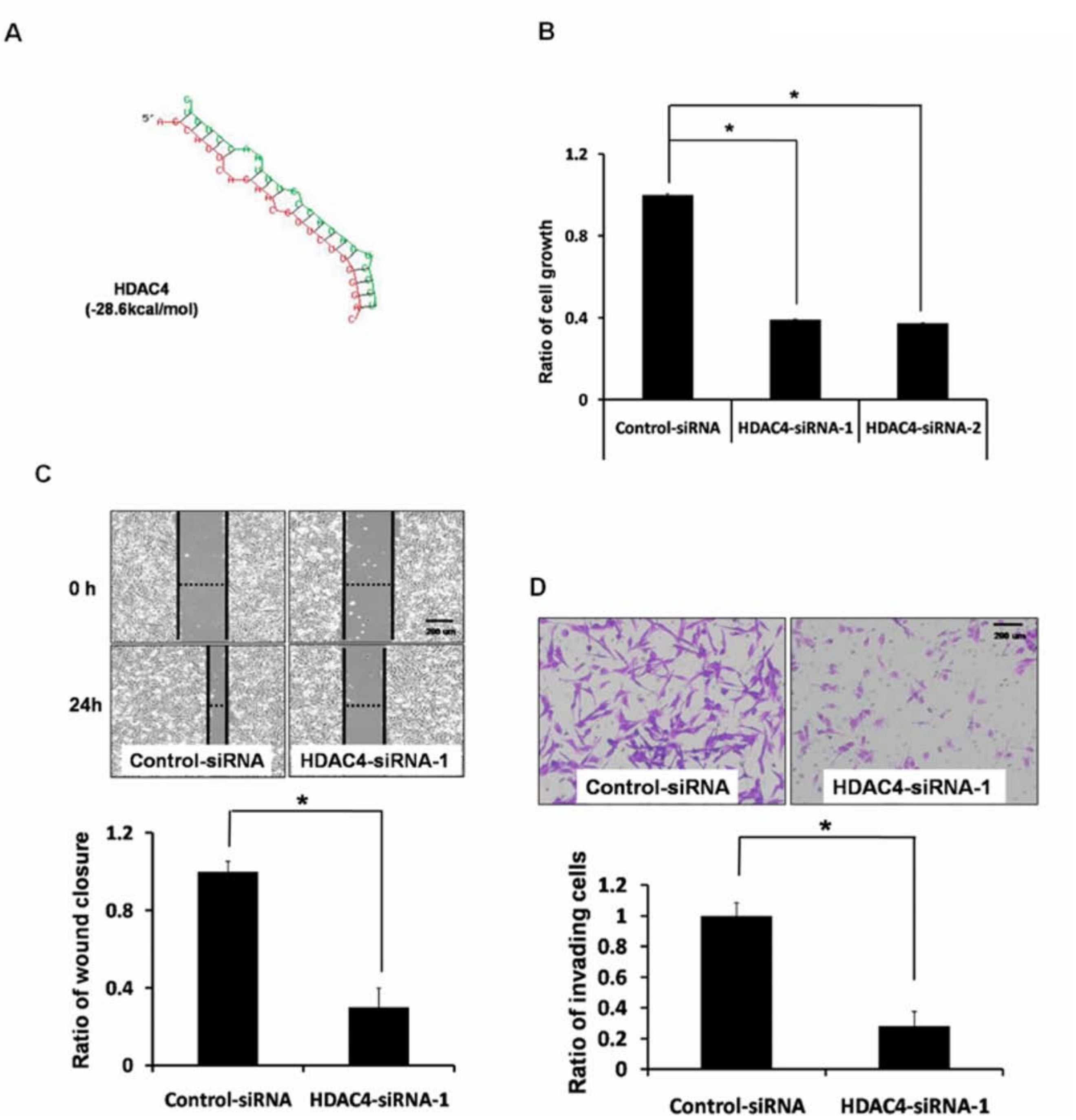

Supplementary Figure S3: HDAC4 promote growth and invasion of MDA-MB-231 breast cancer cells. **(A)** TargetScan was used to predict the miR-125a-5p target site and to calculate the free energy value (−28.6 kcal/mol) of the 3′-UTR of the HDAC4 gene. **(B)** MDA-MB-231 breast cancer cells were transfected with control siRNA or HDAC4 siRNA-1/2 (5 µg) and cell growth was evaluated by counting the cell number 48 hr post-transfection. **(C–D)** MDA-MB-231 cells were transfected as in (B). At the indicated post-transfection times, cell migration was evaluated with wound-healing assay (C). Cell invasion was evaluated with a transwell invasion chamber (D). Data are the means ± SD of three experiments. $*P < 0.05$ *vs*. untreated control determined by two-tailed Student's *t* test. Scale bar = 200 um.

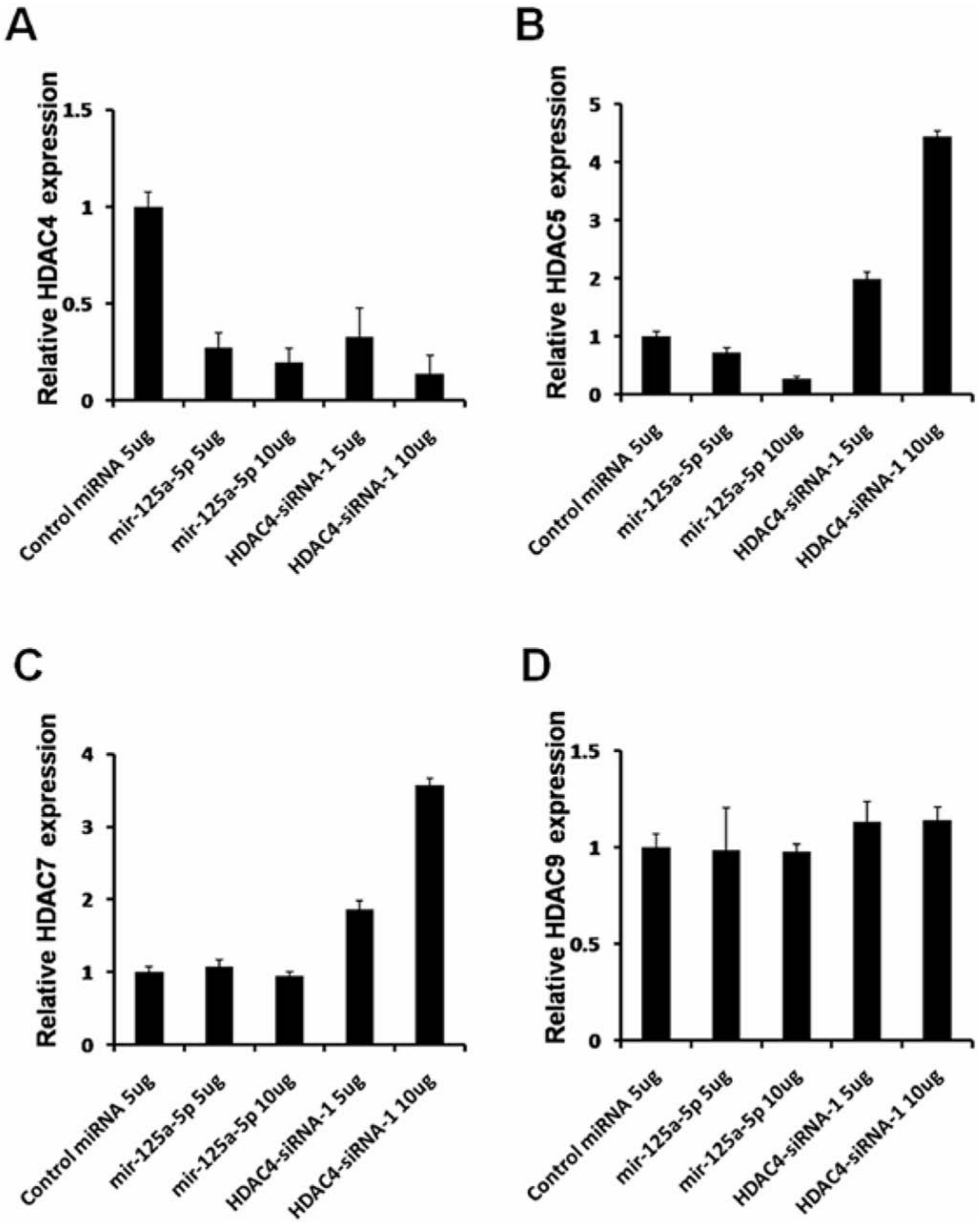

Supplementary Figure S4: Down-regulation of HDAC4 resulted in compensatory increase of classe II HDACs. The R2N1d breast cancer cell line was transfected with control-miRNA (5 µg), miR-125a-5p (5 and 10 µg) or HDAC4-siRNA-1 (5 and 10 µg), and the expression of HDAC4 **(A)**, HDAC 5 **(B)**, HDAC 7 **(C)** and HDAC 9 **(D)** was detected using qRT-PCR 48 hr post-transfection.

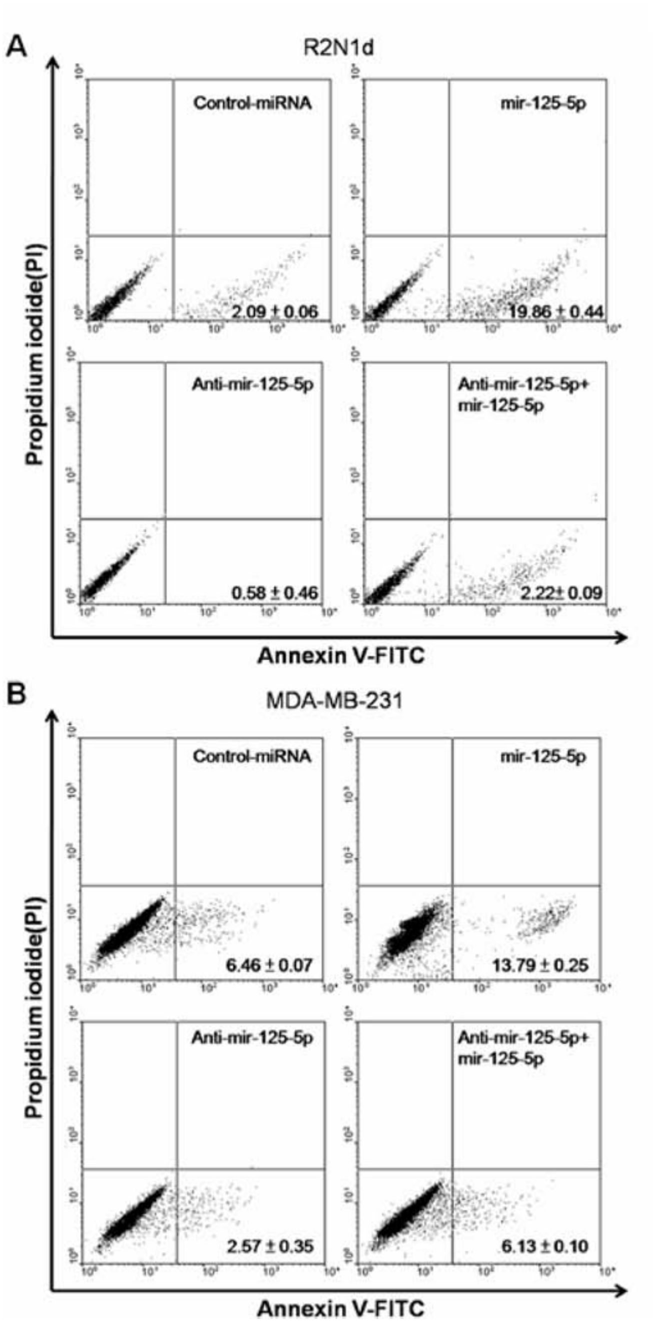

Supplementary Figure S5: miR-125a-5p increases apoptosis in R2N1d and MDA-MB-231 breast cancer cells. (A-B) Breast cancer cells were transfected with control miRNA (5 µg), miR-125a-5p (5 µg), anti-miR-125a-5p (150 nmol/L), or anti-miR-125a-5p (150 nmol/L) + miR-125a-5p (5 µg), and annexin V expression was detected with flow cytometry 24 hr after transfection.

Supplementary Table S1. Clinical information for 10 cases of breast cancer samples with microRNA microarray analysis

Case no	Stage	Grade	LN	ER	PE	Her2/Neu	Tumor size (mm)	Survival status	Survival time (month)
1	1	1	0	1	1	1	20	1	68
2	1	1	0	1	1	1	8	1	61
3	3	3	0	1	1	1	12	1	61
4	1	1	0	1	1	1	6	1	63
5	1	2	0	1	1	1	32	1	66
6	3	3	1	1	1	1	41	0	3
7	3	3	1	1	1	1	25	0	8
8	3	2	1	1	1	1	30	0	6
9	2	2	0	1	1	1	27	0	8
10	2	3	1	1	1	1	36	0	5

Clinical stage: “1” = stage I; “2” = stage II; “3” = stage III
Clinical grade: “1” = grade I; “2” = grade II; “3” = grade III
LN = Lymph-node status: “0” = negative; “1” = positive
ER = Estrogen receptor status: “0” = negative; “1” = positive
PR = Progesterone receptor status: “0” = negative; “1” = positive
Survival status: “0” = death; “1” = survival

Supplementary Table S2. Differentially expressed miRNA in Short survival group *vs*. Long survival group (*n* = 5 in each of the groups)

	Short surviva normalized	SD	Long survival normalized	SD	Fold
hsa-miR-206	1.639979	0.063289	0.067296	0.027301	24.37045
hsa-miR-518a-3p	0.444614	0.070768	0.026207	0.015742	16.96567
hsa-miR-193a-5p	1.911014	0.050979	0.128457	0.06313	14.87707
hsa-miR-155	3.729969	0.038406	0.378776	0.073053	9.84771
hsa-miR-30a	4.958849	3.540145	0.643169	0.146463	7.710235
hsa-miR-503	24.02704	0.073632	3.149682	0.06313	7.628403
hsa-miR-21	0.449407	0.037112	0.094221	0.032472	4.769848
hsa-miR-205	7.816073	0.093429	1.772481	0.048418	4.409802
hsa-miR-194	0.926622	0.865865	0.229429	0.017709	4.038925
hsa-miR-151-3p	0.831575	0.232903	0.260929	0.050918	3.187067
hsa-miR-326	0.466285	0.137389	0.166818	0.071067	2.795254
hsa-miR-300	1.44667	0.204978	0.527609	0.047757	2.74201
hsa-miR-381	144.0849	1.275795	65.11032	0.049056	2.212996
hsa-miR-501-5p	11.90768	0.250637	5.701673	0.082003	2.088511
hsa-miR-34c-3p	5.969289	0.903665	2.917566	0.065777	2.046039
hsa-miR-516a-3p	0.93111	0.155246	0.581309	0.085132	1.601791
hsa-miR-95	0.418476	0.621792	0.27101	0.044476	1.544175
hsa-miR-376a	0.898725	0.182296	0.587203	0.039402	1.53056
hsa-miR-375	0.949318	0.37144	0.673739	0.093377	1.409069
hsa-miR-211	5.588738	0.158547	4.013664	0.04052	1.392467
hsa-miR-523	0.371408	0.16578	0.283593	0.13243	1.309689
hsa-miR-488	1.427643	0.172839	1.157065	0.06118	1.233883
hsa-miR-412	4.46004	0.186638	3.728628	0.067127	1.196194
hsa-miR-508-5p	2.287658	0.767679	2.208276	0.042796	1.035976
hsa-miR-483-5p	0.635428	0.113312	0.619914	0.039715	1.025055
hsa-miR-524-3p	0.961305	0.116048	0.991188	0.075356	0.969879
hsa-miR-188-5p	0.243531	0.082548	0.271742	0.024695	0.896209
hsa-miR-219-1-3p	1.659627	0.312231	1.911259	0.084095	0.868367
hsa-miR-138	0.23517	0.305359	0.31642	0.139627	0.743244
hsa-miR-362-5p	1.642547	0.305212	2.249957	0.063806	0.730055
hsa-miR-139-3p	0.705706	0.218581	1.000825	0.043492	0.705144
hsa-miR-520h	1.95314	0.112102	2.787828	0.045625	0.700615
hsa-miR-320a	0.372482	0.955557	0.55564	0.13276	0.670385
hsa-miR-214	1.011205	0.329239	1.51903	0.075093	0.665709

(*Continued*)

	Short surviva normalized	SD	Long survival normalized	SD	Fold
hsa-miR-320d	1.552892	0.372504	2.516517	0.101184	0.617097
hsa-miR-506	0.185917	0.054375	0.304209	0.026346	0.611168
hsa-miR-493	0.356645	0.124833	0.598878	0.066416	0.595539
hsa-miR-431	0.213893	0.148529	0.370791	0.026918	0.576871
hsa-miR-105	11.10966	0.068339	19.45613	0.068239	0.571026
hsa-miR-339-3p	0.357702	0.044053	0.649122	0.018539	0.55107
hsa-miR-542-5p	6.390032	0.067876	12.03322	0.044814	0.531047
hsa-miR-320c	0.819964	0.442206	1.562265	0.043846	0.52487
hsa-miR-512-3p	0.350453	0.141319	0.681058	0.074785	0.514586
hsa-miR-500	6.421896	0.09807	12.60519	0.054732	0.509479
hsa-miR-181a	1.387851	0.057153	2.877182	0.039865	0.482378
hsa-miR-135a	0.302469	0.179443	0.656907	0.100584	0.460457
hsa-miR-423-3p	0.285033	0.134983	0.632729	0.020599	0.450495
hsa-miR-518b	0.15503	0.206487	0.360127	0.069751	0.430498
hsa-miR-132	82.05304	0.067179	206.7858	0.046415	0.396813
hsa-miR-330-3p	194.228	0.161964	573.4062	0.067437	0.338736
hsa-miR-517a	0.677748	0.231375	2.013195	0.098325	0.336662
hsa-miR-212	0.622623	0.091296	2.056954	0.034985	0.3027
hsa-miR-369-5p	0.409444	0.037631	1.570546	0.041214	0.260709
hsa-miR-181d	0.227236	0.091236	0.877115	0.089049	0.259079
hsa-miR-218	0.124413	0.092748	0.503576	0.200961	0.247067
hsa-miR-486-5p	0.913904	0.056539	4.3673	0.04834	0.209266
hsa-miR-134	0.209869	0.06355	1.25433	0.196924	0.167321
hsa-let-7a	0.159015	0.068696	0.983461	0.018278	0.161693
hsa-let-7b	0.018222	0.045152	0.118745	0.024288	0.153459
hsa-miR-181b	0.08282	0.086324	0.630431	0.042194	0.131374
hsa-miR-520c-3p	0.074183	0.048339	0.625919	0.070519	0.118521
hsa-miR-181c	1.19931	0.077987	10.31172	0.09166	0.116309
hsa-miR-146b-3p	0.201544	0.133459	3.930785	0.063612	0.051275
hsa-miR-125a-5p	0.053726	0.079037	1.949922	0.019964	0.028279

Supplementary Table S3. Clinicopathological characteristics of breast cancer patients

Variables	*N* = 300	Patients (%)
**Stage**		
I	121	40.3
II	133	44.3
III	46	15.3
**Grade**		
I	128	42.7
II	106	35.3
III	66	22.0
**Lymph-node status**		
Negative	177	59.0
Positive	123	41.0
**Estrogen receptor status**		
Negative	101	33.7
Positive	199	66.3
**Progesterone receptor status**		
Negative	125	41.7
Positive	175	58.3
**Her2/Neu status**		
Negative	202	67.3
Positive	98	32.7

Supplementary Table S4. Clinical information of each breast cancer patient

Case no	Median	Stage	Grade	LN	ER	PE	Her2/ Neu	Tumor size (mm)	Survival status	Survival time (month)
1	1	3	3	1	0	0	1	49	0	2
2	0	2	1	0	0	0	0	29	0	2
3	0	2	1	0	1	1	0	23	1	40
4	0	2	2	0	0	0	1	29	1	41
5	0	1	3	1	1	1	0	18	1	41
6	0	1	1	0	1	0	1	23	1	41
7	0	2	2	0	1	1	0	22	1	41
8	0	2	1	0	0	0	1	24	1	37
9	0	2	2	0	0	0	1	50	1	44
10	0	1	2	1	1	0	0	16	1	41
11	0	1	1	1	1	0	0	11	1	42
12	0	1	1	0	1	1	0	8	1	42
13	0	1	2	0	1	0	1	15	1	54
14	0	1	1	0	0	0	0	15	1	54
15	0	1	1	0	0	0	0	20	1	38
16	0	2	2	1	1	0	0	25	1	41
17	1	2	3	1	1	1	0	40	1	43
18	0	1	2	1	1	1	0	9	1	43
19	1	3	3	1	1	0	0	10	1	48
20	0	2	3	1	1	0	0	31	1	43
21	1	1	2	0	1	1	0	11	1	44
22	0	1	2	0	1	1	0	17	1	42
23	0	1	1	0	1	1	0	19	1	44
24	0	1	1	0	1	0	0	15	1	43
25	1	1	1	0	0	0	0	14	1	43
26	0	2	3	1	1	1	1	32	1	64
27	1	1	1	0	0	0	1	15	1	45
28	0	2	2	0	1	0	0	30	1	44
29	0	2	2	1	1	0	0	23	1	42
30	1	1	1	0	1	0	0	18	1	42
31	0	1	2	1	0	0	0	16	1	44
32	0	1	1	1	1	1	0	13	1	44
33	0	3	3	1	0	0	0	53	1	44
34	1	3	2	1	0	0	0	40	1	45
35	1	3	3	1	1	1	1	32	1	56
36	1	1	1	0	0	0	0	32	1	46

(*Continued*)

Case no	Median	Stage	Grade	LN	ER	PE	Her2/ Neu	Tumor size (mm)	Survival status	Survival time (month)
37	0	3	2	0	0	0	0	60	1	43
38	1	2	2	1	1	0	0	32	1	45
39	1	1	1	0	1	0	0	13	1	57
40	0	2	2	0	1	1	0	21	1	45
41	1	1	1	0	1	1	0	15	1	44
42	1	2	3	1	1	1	1	23	1	44
43	1	1	1	0	0	1	0	18	1	44
44	1	2	2	0	0	0	1	21	1	46
45	1	1	1	0	0	0	1	34	1	58
46	1	1	1	0	1	0	0	36	1	45
47	1	2	2	0	0	0	1	24	1	46
48	1	2	2	1	1	1	0	29	1	47
49	1	1	1	0	0	1	0	10	1	45
50	1	1	2	0	1	1	1	19	1	46
51	1	2	1	0	1	1	1	27	1	46
52	1	2	2	0	1	1	1	22	1	47
53	1	2	1	0	1	1	1	22	1	45
54	1	2	1	0	1	0	1	24	1	47
55	0	2	2	0	1	1	1	27	1	46
56	0	1	1	0	0	1	1	10	1	47
57	1	1	1	0	1	1	0	13	1	41
58	0	3	2	1	1	1	0	8	1	48
59	0	2	2	1	0	0	1	37	1	47
60	1	3	3	0	1	1	1	30	1	54
61	0	3	3	1	1	1	1	110	0	32
62	0	1	1	0	1	1	0	14	1	48
63	1	1	1	0	0	0	0	35	1	44
64	0	2	2	0	0	0	0	35	1	47
65	1	2	2	0	1	1	1	40	1	69
66	0	1	2	0	0	0	0	19	1	48
67	0	2	2	0	0	0	0	35	1	48
68	1	3	3	0	1	1	0	12	1	51
69	1	1	1	0	0	0	1	39	1	50
70	1	1	3	1	1	1	0	15	1	47
71	1	2	2	1	0	0	1	23	1	49
72	0	1	1	0	0	0	0	13	1	60
73	0	2	2	0	1	1	0	30	1	48

(*Continued*)

Case no	Median	Stage	Grade	LN	ER	PE	Her2/ Neu	Tumor size (mm)	Survival status	Survival time (month)
74	0	2	1	0	1	0	0	31	1	48
75	1	2	2	1	0	0	0	40	0	30
76	0	1	1	0	1	1	0	15	1	49
77	1	1	1	0	1	1	0	15	1	49
78	1	1	1	0	1	1	0	18	1	48
79	0	2	2	0	0	0	1	28	1	56
80	1	3	2	0	0	0	0	35	0	40
81	0	1	1	0	1	0	0	7	1	48
82	1	2	1	0	0	0	0	34	1	48
83	0	1	1	0	0	0	1	19	1	49
84	1	2	2	0	0	0	0	23	1	50
85	1	2	3	1	1	1	0	26	1	48
86	0	2	2	1	0	0	1	21	1	49
87	0	1	1	0	1	1	0	18	1	49
88	1	2	2	0	1	1	0	25	1	62
89	1	1	2	1	1	1	0	15	1	50
90	0	1	1	0	1	1	0	15	1	50
91	1	2	2	0	1	1	0	26	1	50
92	0	2	2	0	1	1	1	43	1	48
93	0	2	2	1	1	1	0	37	1	62
94	0	2	2	1	1	0	1	22	1	63
95	1	3	3	1	1	1	0	24	1	50
96	0	3	3	1	0	1	0	80	1	46
97	1	1	1	0	1	1	1	12	1	51
98	1	1	1	0	0	0	0	15	1	50
99	1	1	1	0	0	0	1	9	1	50
100	0	3	1	0	1	1	0	25	1	50
101	1	2	2	0	1	1	0	25	1	49
102	0	1	1	0	1	1	1	21	1	51
103	1	2	3	1	1	1	0	22	1	51
104	1	1	1	0	1	1	0	10	1	52
105	1	2	1	0	1	0	0	22	1	49
106	1	1	1	0	0	0	0	18	1	50
107	1	2	2	0	1	1	0	45	1	50
108	1	2	2	1	0	0	1	38	1	51
109	1	2	3	1	1	1	0	23	1	51
110	1	2	2	1	1	1	1	22	1	50

(*Continued*)

Case no	Median	Stage	Grade	LN	ER	PE	Her2/ Neu	Tumor size (mm)	Survival status	Survival time (month)
111	1	2	3	1	0	0	0	33	1	53
112	0	1	1	0	1	1	0	13	1	53
113	0	2	3	1	1	1	0	35	1	53
114	1	1	1	0	1	1	0	20	1	52
115	1	1	1	0	1	1	0	43	1	65
116	1	2	1	0	0	1	0	24	1	53
117	0	1	1	0	1	1	0	40	1	53
118	0	1	1	0	1	1	1	15	1	65
119	0	3	3	1	1	1	1	44	1	55
120	0	2	2	0	1	0	0	30	1	53
121	1	2	2	1	0	0	0	22	0	21
122	0	3	2	1	1	1	0	40	1	53
123	1	3	3	1	0	0	1	30	1	51
124	0	1	3	1	0	0	1	14	1	52
125	0	2	1	0	0	0	1	30	1	53
126	1	2	1	0	1	1	1	30	1	54
127	1	2	1	0	1	1	1	23	1	53
128	0	2	2	0	1	1	0	30	1	52
129	0	3	3	0	0	0	0	40	1	68
130	1	1	1	0	1	1	0	15	1	52
131	0	3	3	1	0	0	1	31	0	32
132	0	3	1	0	1	1	1	60	1	53
133	0	1	1	0	1	1	0	53	1	54
134	1	1	1	0	1	1	1	15	1	52
135	0	1	1	0	0	0	0	13	1	55
136	1	2	3	0	1	1	1	30	1	54
137	1	2	2	0	1	1	0	28	1	53
138	1	1	1	0	1	1	0	10	1	54
139	1	1	1	0	1	1	0	20	1	53
140	0	2	1	0	0	0	1	30	1	53
141	0	1	3	1	1	1	1	16	1	54
142	0	1	1	0	1	1	1	7	1	53
143	1	2	2	1	1	1	1	26	1	66
144	0	1	1	0	1	1	1	15	1	54
145	0	1	1	0	1	1	1	12	1	54
146	1	1	3	1	1	0	1	20	1	54

(*Continued*)

Case no	Median	Stage	Grade	LN	ER	PE	Her2/ Neu	Tumor size (mm)	Survival status	Survival time (month)
147	1	1	1	0	1	1	1	17	1	54
148	0	3	2	0	1	1	1	50	1	54
149	0	1	1	0	1	1	0	25	1	53
150	0	2	2	0	1	0	0	22	1	55
151	0	2	2	1	0	0	1	29	1	55
152	1	1	1	0	1	1	0	15	1	55
153	0	3	3	1	0	0	1	22	1	54
154	0	1	2	1	1	1	0	14	0	40
155	0	3	1	0	1	0	0	50	1	56
156	1	2	3	1	1	0	1	34	1	56
157	1	2	1	1	1	1	0	23	1	56
158	0	1	1	0	0	0	0	20	1	55
159	0	3	1	0	1	1	0	60	1	57
160	1	2	1	0	1	1	1	23	1	56
161	1	3	3	1	0	0	0	5	0	15
162	1	1	1	0	1	1	1	14	1	56
163	0	3	3	0	0	0	1	100	1	59
164	0	1	1	0	0	0	0	70	1	58
165	0	2	1	0	0	0	0	30	1	57
166	0	1	1	0	0	0	0	20	1	69
167	1	2	2	0	1	1	0	40	1	55
168	1	1	1	0	0	1	0	20	1	56
169	0	2	2	0	0	0	0	25	1	69
170	0	1	1	0	1	1	0	15	1	57
171	1	1	1	0	0	0	0	19	1	57
172	1	3	2	1	1	0	1	21	1	57
173	1	1	1	0	0	0	1	9	1	56
174	0	2	1	0	1	1	1	34	1	57
175	0	3	3	1	1	1	0	100	1	58
176	1	1	1	0	1	1	0	13	1	57
177	0	1	2	1	1	1	0	19	1	58
178	0	2	1	0	0	0	0	21	1	70
179	0	2	2	0	0	0	0	30	1	58
180	0	1	1	0	1	1	0	30	1	57
181	0	2	2	0	1	1	1	30	1	58
182	1	1	1	0	0	0	0	10	1	58
183	1	2	2	1	1	1	0	21	1	58

(*Continued*)

Case no	Median	Stage	Grade	LN	ER	PE	Her2/ Neu	Tumor size (mm)	Survival status	Survival time (month)
184	1	1	1	0	1	1	0	15	1	58
185	0	1	2	1	1	1	0	20	1	57
186	0	3	3	1	1	1	1	150	1	61
187	0	1	1	0	1	1	0	10	1	57
188	0	1	1	0	1	1	0	15	1	56
189	0	2	1	0	1	1	1	32	1	59
190	1	1	1	0	0	0	0	12	1	56
191	1	3	1	0	1	1	0	60	1	61
192	1	2	1	0	1	1	0	21	1	59
193	1	1	1	0	1	1	0	2	1	70
194	1	2	2	0	0	0	1	30	1	53
195	1	2	1	0	1	1	0	22	1	57
196	1	2	2	0	0	1	1	23	1	57
197	1	2	3	1	1	1	0	29	1	57
198	1	1	2	0	0	0	0	20	1	59
199	1	1	1	0	0	0	1	15	1	59
200	1	1	3	1	0	0	0	20	1	59
201	1	1	1	0	0	0	1	30	1	59
202	1	1	1	0	1	1	0	15	1	53
203	1	1	1	0	1	0	1	20	1	58
204	1	1	1	0	1	1	1	20	1	60
205	1	2	3	1	0	0	1	32	0	45
206	1	2	2	1	0	0	0	21	1	60
207	0	2	1	0	1	0	1	30	1	59
208	1	1	3	1	0	0	0	10	1	60
209	1	1	1	0	1	1	0	13	1	60
210	0	3	2	1	0	0	0	30	1	61
211	1	1	1	0	1	1	0	20	0	50
212	1	2	2	0	1	1	0	25	1	55
213	1	2	2	0	0	1	0	8	1	60
214	1	2	3	1	0	0	1	20	1	61
215	0	2	2	0	1	0	0	27	0	38
216	0	3	2	1	1	0	0	30	0	42
217	0	1	1	0	1	1	0	11	1	57
218	0	3	3	1	1	1	0	37	1	61
219	0	2	1	0	1	1	0	41	1	61
220	1	2	1	0	1	1	0	25	1	60
221	1	1	1	0	1	0	1	20	1	62

(*Continued*)

Case no	Median	Stage	Grade	LN	ER	PE	Her2/ Neu	Tumor size (mm)	Survival status	Survival time (month)
222	1	2	3	1	1	1	1	31	1	60
223	1	2	1	0	1	1	1	40	1	74
224	1	2	2	0	0	0	0	22	1	61
225	1	2	1	0	1	1	0	30	1	61
226	1	2	2	1	0	0	1	28	1	61
227	1	1	1	0	0	0	0	15	1	58
228	1	1	1	0	0	0	1	20	0	50
229	0	2	2	0	1	1	0	37	1	74
230	1	2	1	0	1	1	0	22	1	62
231	1	3	2	0	0	0	0	33	1	61
232	1	3	1	0	1	1	1	37	1	62
233	1	1	1	0	0	0	1	19	1	63
234	1	2	2	1	1	1	0	10	1	61
235	1	3	2	1	1	1	1	30	1	62
236	1	1	1	0	0	0	1	20	1	61
237	1	1	1	0	1	0	0	12	1	61
238	1	1	1	0	1	1	1	9	1	58
239	1	1	2	1	1	1	0	20	1	63
240	0	3	3	1	0	0	0	32	0	32
241	1	1	1	0	1	1	0	17	1	61
242	0	2	1	0	1	1	0	40	1	63
243	1	1	1	1	0	0	0	15	1	62
244	1	1	1	0	1	1	0	11	1	62
245	0	2	1	0	1	1	1	28	1	63
246	1	2	1	0	1	1	1	20	1	63
247	1	2	2	0	0	0	0	40	1	60
248	1	3	2	1	1	1	0	25	1	63
249	0	2	1	0	1	1	0	25	1	64
250	0	1	2	0	0	0	0	14	1	65
251	1	2	3	1	1	1	1	10	1	63
252	1	2	3	1	1	1	1	32	1	64
253	0	2	3	1	1	1	0	28	1	24
254	0	2	3	1	1	1	0	35	1	24
255	1	2	2	1	1	1	0	35	1	24
256	1	2	2	1	1	1	0	30	1	24
257	1	2	1	1	1	1	0	24	1	24
258	0	2	3	1	1	1	0	39	1	24
259	0	2	2	1	0	0	0	21	1	24

(*Continued*)

Case no	Median	Stage	Grade	LN	ER	PE	Her2/ Neu	Tumor size (mm)	Survival status	Survival time (month)
260	0	1	3	1	1	1	0	35	1	25
261	0	2	2	1	1	1	0	21	1	24
262	0	2	2	1	1	1	0	30	1	25
263	0	2	3	1	1	1	0	15	1	25
264	0	2	2	1	1	1	0	30	1	25
265	0	1	2	1	0	0	0	10	1	25
266	0	1	2	1	0	0	0	18	1	25
267	0	1	2	1	1	1	0	18	1	25
268	0	1	3	1	1	1	0	24	1	25
269	0	2	2	1	0	0	0	21	1	26
270	0	1	3	1	0	0	0	10	1	26
271	0	2	2	1	1	1	0	28	1	25
272	0	2	3	1	1	1	0	19	1	26
273	0	2	2	1	1	1	0	7	1	26
274	0	2	3	1	1	1	0	47	1	26
275	0	2	3	1	1	1	0	42	1	25
276	0	1	2	1	1	1	0	15	1	26
277	0	1	2	1	1	1	0	17	1	26
278	0	2	3	1	1	1	0	30	1	26
279	0	1	3	1	1	1	0	20	1	27
280	0	1	2	1	1	1	0	15	1	26
281	0	2	3	1	1	1	0	35	1	26
282	0	2	2	1	1	1	0	30	1	26
283	0	2	2	1	0	0	0	25	1	27
284	0	2	2	1	1	1	0	30	1	27
285	0	2	3	1	1	1	0	29	1	27
286	0	3	3	0	0	0	0	67	0	16
287	0	2	2	1	1	1	1	60	0	4
288	0	3	3	1	0	0	1	33	0	1
289	0	3	3	1	1	0	0	45	0	3
290	0	3	3	1	0	0	0	12	0	7
291	0	1	1	0	0	0	1	5	0	7
292	0	2	2	1	1	1	0	200	0	7
293	0	3	3	1	1	1	1	110	0	2
294	0	3	3	0	0	0	0	27	0	9
295	0	2	2	1	1	1	0	70	0	3

(*Continued*)

Case no	Median	Stage	Grade	LN	ER	PE	Her2/ Neu	Tumor size (mm)	Survival status	Survival time (month)
296	0	2	2	1	1	1	0	100	0	8
297	0	2	2	1	1	1	1	30	0	8
298	0	3	3	1	0	0	0	35	0	9
299	0	2	2	1	1	1	1	45	0	1
300	0	3	3	1	1	1	1	100	0	0

Median = mir-125a-5p expression median:"0" = ≤ median; "1" = > median
Clinical stage: "1" = stage I; "2" = stage II; "3" = stage III
Clinical grade: "1" = grade I; "2" = grade II; "3" = grade III
LN = Lymph-node status: "0" = negative; "1" = positive
ER = Estrogen receptor status: "0" = negative; "1" = positive
PR = Progesterone receptor status: "0" = negative; "1" = positive
Survival status: "0" = death; "1" = survival

Supplementary Table S5. The percentage of tumor development for animal experiments

	1×10^3	1×10^5	1×10^7
R2N1d-YFP	6/6	6/6	6/6
R2N1d-GFP-miR-125a-5p	0/6	0/6	2/6
